# Characterization of Ghrelin O-Acyltransferase (GOAT) in goldfish (*Carassius auratus*)

**DOI:** 10.1371/journal.pone.0171874

**Published:** 2017-02-08

**Authors:** Ayelén Melisa Blanco, Miguel Gómez-Boronat, Ángel Luis Alonso-Gómez, Roman Yufa, Suraj Unniappan, María Jesús Delgado, Ana Isabel Valenciano

**Affiliations:** 1 Departamento de Fisiología (Fisiología Animal II), Facultad de Biología, Universidad Complutense de Madrid, Madrid, Spain; 2 Department of Biology, York University, Toronto, Ontario, Canada; 3 Laboratory of Integrative Neuroendocrinology, Department of Veterinary Biomedical Sciences, Western College of Veterinary Medicine, University of Saskatchewan, Saskatoon, Saskatchewan, Canada; Karlsruher Institut fur Technologie, GERMANY

## Abstract

Ghrelin is the only known hormone posttranslationally modified with an acylation. This modification is crucial for most of ghrelin’s physiological effects and is catalyzed by the polytopic enzyme ghrelin O-acyltransferase (GOAT). The aim of this study was to characterize GOAT in a teleost model, goldfish (*Carassius auratus*). First, the full-length cDNA sequence was obtained by RT-PCR and rapid amplification of cDNA ends methods. Two highly homologous cDNAs of 1491 and 1413 bp, respectively, named *goat-V1* and *goat-V2* were identified. Deduced protein sequences (393 and 367 amino acids, respectively) are predicted to present 11 and 9 transmembrane regions, respectively, and both contain two conserved key residues proposed to be involved in catalysis: asparagine 273 and histidine 304. RT-qPCR revealed that both forms of *goat* mRNAs show a similar widespread tissue distribution, with the highest expression in the gastrointestinal tract and gonads and less but considerable expression in brain, pituitary, liver and adipose tissue. Immunostaining of intestinal sections showed the presence of GOAT immunoreactive cells in the intestinal mucosa, some of which colocalize with ghrelin. Using an *in vitro* approach, we observed that acylated ghrelin downregulates GOAT gene and protein levels in cultured intestine in a time-dependent manner. Finally, we found a rhythmic oscillation of *goat* mRNA expression in the hypothalamus, pituitary and intestinal bulb of goldfish fed at midday, but not at midnight. Together, these findings report novel data characterizing GOAT, and offer new information about the ghrelinergic system in fish.

## Introduction

Ghrelin O-acyltransferase (GOAT) is a member of the membrane-bound O-acyltransferase (MBOAT) family [1], previously known as MBOAT4 [2–5]. It is responsible for the attachment of a medium-chain fatty acid, typically octanoate, to the third aminoacid (a serine residue) of the N-terminal region of ghrelin, a peptide hormone mainly synthesized by the gut [6]. This posttranslational acyl modification of ghrelin is essential for the peptide to bind to its receptor, the growth hormone secretagogue receptor (GHS-R), and so to exert most of its physiological functions [6]. This includes a role in a wide variety of physiological processes of all vertebrates, including fish, such as stimulation of growth hormone release from the pituitary, stimulation of food intake, carbohydrate utilization and adiposity and regulation of insulin secretion, among others [7–11]. In the last decade, ghrelin has also been involved in the circadian system of both mammals and fish, where it might act as both an input [12–14] and an output signal [15]. Although acylated ghrelin (acyl-GRL) is typically considered as the active form of the hormone, studies have recently shown that deacylated ghrelin (desacyl-GRL) also has some biological functions in vertebrates [16–18].

The identification of GOAT occurred in 2008 by two independent research groups who simultaneously reported the identification of the enzyme in mammals [19,20]. GOAT shares structural similarities with other members of the MBOAT family. Indeed, 16 MBOAT protein sequences have been identified from the mouse genome, and 11 putative catalytic domains are highly conserved among all the sequences, as well as the residues asparagine 307 (Asn-307) and histidine 338 (His-338) that are essential for the catalytic activity of the enzymes [20,21]. The GOAT sequence is also highly conserved across vertebrates from zebrafish to humans, with a 90% homology for human and rodent, and 59% for mammalian and zebrafish [19,22].

GOAT seems to be located in the membrane of endoplasmic reticulum and its topology in vertebrates was recently described to comprise 11 transmembrane domains (TMDs) and one reentrant loop connected by 11 exposed-loop regions [23]. The acylation process of ghrelin by GOAT occurs after the signal sequence from pre-pro-ghrelin is cleaved by a signal peptide peptidase to form pro-ghrelin, which can be produced either as acylated or unacylated pro-ghrelin [4,24]. Acylated pro-ghrelin is produced when GOAT in the endoplasmic reticulum translocates mainly octanoyl-CoAs from the cytosolic side to acylate the precursor. In this regard, GOAT has been postulated to be a possible acyl-CoA transporter. Ghrelin-derived peptides as short as four amino acids can be acyl-modified by GOAT [25], suggesting that it is likely that GOAT recognizes a N-terminal four-amino acids motif within the intact pro-ghrelin peptide. Once in the Golgi, pro-protein convertases 1/3 and 2 cleave acylated or unacylated pro-ghrelin to form mature forms of ghrelin, which are packaged into vesicles and released into the circulation [4,24]. Although mature ghrelin can be synthesized as both acyl-GRL and desacyl-GRL, ghrelin can also be deacylated by the acyl-protein thioesterase 1/lisophospholipase I mainly released from the liver [26].

In rodents, the highest levels of GOAT mRNAs are found in the stomach and intestine followed by the testis [20], and pancreas [27]. GOAT was found to be localized within the ghrelin producing cells in stomach and duodenum of mice and rats [28,29]. Interestingly, it was estimated that about 15% of the GOAT expressing cells in the gastric mucosa did not coexpress ghrelin, suggesting the existence of other endogenous substrate for this enzyme. In humans, Gutierrez and coworkers [19] demonstrated that *goat* transcript levels are more abundant in stomach and pancreas, whereas Lim and coworkers [30] found the highest expression in adrenal cortex, spleen and lung. Expression of *goat* in mammalian tissues was also described in the hypothalamus, pituitary, taste cells, heart, kidney, adipose tissue, ovary, placenta, muscle, serum and chondrocytes [28,31–33]. Recently, the tissue expression pattern of *goat* has been described in zebrafish, where high levels of mRNA expression were observed in gut, brain and gonads [22].

The importance of ghrelin acylation for the functioning of the ghrelinergic system in all vertebrates, including fish [18], led us to characterize GOAT in a teleost model, the goldfish (*Carassius auratus*). To achieve that purpose, we first obtained the full-length cDNA sequence of GOAT, and we carried out both an *in silico* structural and phylogenetic analyses of the sequence obtained. Second, we characterized the distribution of the enzyme mRNAs in central and peripheral tissues, as well as its distribution in the intestine in relation to its substrate ghrelin. Third, we studied the possible modulation of GOAT by ghrelin using a primary intestinal culture system. Finally, considering the recent proposal of a crosstalk between ghrelin and the circadian system [34] and the fact that daily expression rhythms have been described for ghrelin and its main receptor in goldfish [15], we studied the daily expression profile of *goat* in central and peripheral tissues of goldfish. Given the relevance of the ghrelinergic system on feeding [9,35], we also determined the possible influence of feeding time on such daily profiles.

## Materials and methods

### Animals and sampling

Goldfish with a body weight of 27 ± 7 g were obtained from a local commercial supplier in Madrid (Madrid, Spain) for all the studies, except for the immunohistochemistry and *in vitro* assays for which goldfish were obtained from a commercial supplier in Calgary (Alberta, Canada). Fish were housed in 60 L aquaria (n = 10/aquarium) with filtered fresh water (21 ± 2°C) and continuous aeration, and maintained under a 12 h of light: 12 h of darkness (12L:12D) photoperiod (lights on at 08:00 h). Food from a commercial flake diet (1% body weight, Sera Pond, Heinserberg, Germany) was offered daily at 10:00 h for at least two weeks before the assays. For the experimental procedures, fish were anesthetized with tricaine methanesulfonate (MS-222, Sigma-Aldrich, Madrid, Spain) and sacrificed. Sample collection is detailed below for each study. All procedures carried out at the University of Madrid were approved by the Animal Experimentation Committee of Complutense University, and performed according to the European Communities Council Directive (2010/63/UE). For the studies conducted at the University of Saskatchewan, all animal use and care strictly followed the regulations of the Canadian Council for Animal Care and were approved by the institutional animal research ethics committee (Protocol # 2012–0082).

### Identification of GOAT cDNA sequence

To obtain a partial sequence of goldfish *goat*, one fish was sacrificed at 11:00 h, no feeding the sampling day, and the intestinal bulb was collected. Total RNA was isolated using TRI Reagent (Sigma-Aldrich, Madrid, Spain) and treated with RQ1 RNase-Free DNase (Promega, Madison, USA) according to the manufacturer’s instructions. Then, an aliquot of 1 μg of total RNA was reverse transcribed into cDNA in a 25 μL reaction volume using random primers, RNase inhibitor and SuperScript II Reverse Transcriptase (Invitrogen, Carlsbad, USA). The reverse transcription reaction conditions consisted in 25°C for 10 min, an extension of 50 min at 42°C and a denaturalization step at 70°C for 15 min. The first strand cDNA fragments obtained were used as a template to amplify *goat* using various sets of primers (Table 1), all obtained from Sigma-Aldrich. Primers were hand-made designed by selecting convergent parts between the enzyme nucleotide sequence of *Danio rerio* (GenBank ID: NM_001122944.1) and *Astyanax mexicanus* (Ensembl ID: ENSAMXT00000014838) using an alignment tool (http://www.ebi.ac.uk/Tools/msa/clustalw2/). The PCRs were performed in a 25 μl reaction volume containing 1.25 U of *Taq*DNA Polymerase recombinant, PCR Buffer (20 mM Tris-HCl pH 8.4, 50 mM KCl), 1.5 mM of MgCl_2_, 0.2 mM of dNTP mixture (all from Invitrogen), 0.2 μM of each forward and reverse primer and 1–2 μl of cDNA. Reaction conditions underwent an initial incubation at 94°C for 3 min, followed by 40 cycles of 94°C for 45 sec, 57°C/60°C for 30 sec and 72°C for 1 min, with a final extension step at 72°C for 10 min. For some reactions, touchdown PCRs were performed. PCR products were electrophoresed on a 2% agarose gel. Single bands for each PCR were purified using GenElute^™^ Gel Extraction Kit (Sigma-Aldrich) and sequenced (Secugen, Madrid, Spain). The nucleotide deduced sequences were analyzed with the BLAST program on the National Center for Biotechnology Information (NCBI) website (http://blast.ncbi.nlm.nih.gov/).

**Table 1:** Primers designed for cloning and expression analysis of *goat*.

Primer name	Sequence (5’ to 3’)	Application
GOAT Forward 1	GCTATTGCAACAATGGGTCCATA	Partial cloning
GOAT Forward 2	CTGCAGATGTGTTGGCAAAC	Partial cloning
GOAT Forward 4	ATTGCTGTTCTTCAGTGCCG	RT-qPCR
GOAT Forward 5	TGATGACACAGAGGGTTTCTTCTC	Full-length sequence
GOAT Forward 6	TCTCTTGATCTGCAGGAAGGAAC	Full-length sequence
GOAT Forward 7	TTGAGTCGTCCTTCAGCGTTT	Full-length sequence
GOAT Forward 8	GGCCTTCATCCAGGTCAGATTC	Full-length sequence
GOAT Forward 9	CTGGTCTGTCACCGTGCAA	Full-length sequence
GOAT Forward 11	CACTCCAGTAAGGTCTGTTGG	Full-length sequence
GOAT Forward 13	TCCAGTAAGGTCTGTTGGCAC	Full-length sequence
GOAT Forward V1	CACTGGGAGGATTCATCTTTGC	RT-qPCR
GOAT Forward V2	TCAGAATCTGTGCCGTCCTT	RT-qPCR
GOAT Reverse 2	TCTTGAAGCCAGTATATCTGGTACTG	Partial cloning, Full-length sequence
GOAT Reverse 3	GCAAAGTGGACCTCCAAGAAG	Partial cloning, Full-length sequence
GOAT Reverse 5	TGTACAAGTGCCAGACGGTT	Full-length sequence, RT-qPCR
GOAT Reverse 6	GCAGGGAAATAAAGCGAGTAGGT	Partial cloning, Full-length sequence
GOAT Reverse 8	CGGCACTGAAGAACAGCAATAAG	Full-length sequence
GOAT Reverse 10	GAAGCACTGCACTGAAGAACA	Full-length sequence
GOAT Reverse 12	TGATGCATACAGAGCACATT	Full-length sequence
GOAT Reverse 14	GCTTGATGCATACAGAGCACA	Full-length sequence
GOAT Reverse 15	GCAAGCTTGGTTAAGCTTTCATGT	Full-length sequence
GOAT Reverse V1	GCAGTCCCAGTGTCCAATGA	RT-qPCR
GOAT Reverse V2	TGGTGTCTCTTGAAGCCAGT	RT-qPCR
Abridged Anchor Primer	GGCCACGCGTCGACTAGTACGGGIIGGGIIGGGIIG	5’ RACE
Universal Amplification Primer	CUACUACUACUAGGCCACGCGTCGACTAGTAC	5’ RACE, 3’ RACE
Adapter Primer	GGCCACGCGTCGACTAGTACTTTTTTTTTTTTTTTTT	3’ RACE
β-Actin Forward	CTACTGGTATTGTGATGGACT	RT-qPCR
β-Actin Reverse	TCCAGACAGAGTATTTGCGCT	RT-qPCR
EF-1α Forward	CCCTGGCCACAGAGATTTCA	RT-qPCR
EF-1α Reverse	CAGCCTCGAACTCACCAACA	RT-qPCR

In order to obtain the *goat* full-length sequence, rapid amplification of the cDNA ends (3’ RACE and 5’ RACE) was performed using commercial kits (Invitrogen). PCRs were carried out using adaptor primers provided with the kits and gene-specific primers designed from the partial cDNA sequence of goldfish GOAT already obtained (Table 1). Reactions were performed in a 50 μl final volume containing 1.25 U of *GoTaq* Hot Start Polymerase, PCR Buffer, 2 mM of MgCl_2_ (all from Promega), 0.2 mM of dNTP mixture (Invitrogen), 0.2 μM of each forward and reverse primer and 2 μl of cDNA. Cycling conditions comprised an initial incubation at 94°C for 3 min, followed by 30 cycles of 94°C for 45 sec, 57°C for 30 sec and 72°C for 30 sec, with a final extension step at 72°C for 5 min. The PCR products were electrophoresed on a 1.5% agarose gel and target bands for each PCR were purified and sequenced as described above. The full-length *goat* cDNA sequence was obtained by assemblage using the DNA Baser software. Sequencing of *goat* PCR products revealed the existence of two highly homologous cDNAs that were named goldfish *goat-V1* and goldfish *goat-V2*.

The possible presence of introns within the nucleotide sequences was analyzed carrying out PCRs with genomic DNA. Genomic DNA was isolated from Trizol samples after the RNA-containing aqueous phase had been removed. PCRs were performed as above described using *GoTaq* Hot Start Polymerase (Promega) and PCR products were purified and sequenced. The nucleotide sequences retrieved from the DNA sequencing service were aligned with the full-length GOAT DNA sequence obtained using a web aligning tool (http://www.ebi.ac.uk/Tools/msa/clustalw2/) to identify the presence of introns.

### *In silico* structural and phylogenetic analyses

An *in silico* structural analysis of both GOAT sequences obtained was carried out using various online bioinformatics tools. The predictions of protein molecular weight and other basic properties were carried out using the ProtParam tool from Expasy (http://web.expasy.org/protparam/). The presence and sequence of signal peptide were predicted using SignalP 4.1 (http://www.cbs.dtu.dk/services/SignalP/). Topology prediction servers, including DAS (http://www.sbc.su.se/~miklos/DAS/), MemBrain (http://www.csbio.sjtu.edu.cn/bioinf/MemBrain/), MEMSAT-SVM (http://bioinf.cs.ucl.ac.uk/psipred/), Phobius (http://phobius.sbc.su.se/), PredictProtein (http://ppopen.informatik.tu-muenchen.de/), Protter (http://wlab.ethz.ch/protter/), SOSUI (http://harrier.nagahama-i-bio.ac.jp/sosui/sosui_submit.html), TMHMM (http://www.cbs.dtu.dk/services/TMHMM/), TMPred (http://www.ch.embnet.org/software/TMPRED_form.html) and TopPred 1.10 (http://mobyle.pasteur.fr/cgi-bin/portal.py?#forms::toppred), were used to predict the topological organization of the enzyme. Finally, a phylogenetic analysis was performed by aligning the goldfish GOAT amino acid sequences with those of other vertebrates retrieved from GenBank and Ensembl Genome using the Clustal-W2 tool (http://www.ebi.ac.uk/Tools/msa/clustalw2/). A phylogenetic tree was constructed by the neighbor-joining method, with 1000 replicates for the bootstrap test, using MEGA6 [36]. The elephant shark (*Callorhinchus milii*) was considered as outgroup in the phylogenetic analysis given the basal phylogenetic position of cartilaginous fish and their relevance in the study of the evolution of genes and gene regulation in vertebrates.

### Tissue distribution of *goat* mRNA using RT-qPCR

The expression pattern of the two identified splice variants of *goat* in goldfish tissues was studied using real-time reverse transcription-quantitative PCR (RT-qPCR). Samples of brain, pituitary, head kidney, gills, heart, esophagus, intestinal bulb, anterior intestine, middle intestine, posterior intestine, liver, spleen, kidney, gonads, adipose tissue and muscle (n = 4) were collected. Total RNA extraction and reverse transcription of 2 μg of RNA were performed as above described. Then, a real-time or quantitative PCR (qPCR) was performed using iTaq^ℒ^ Universal SYBR^®^ Green Supermix (Bio-Rad, Hercules, USA). The specific primers sequences used for target gene *goat* and reference gene *elongation factor 1α* (*ef-1α*; accession number AB056104) were ordered to Sigma-Aldrich and are shown in Table 1. Each set of primers for *goat* were designed to be specific for one of the splice variants and unable to amplify the other one (primers GOAT Forward and Reverse V1 from Table 1 were used to amplify the first variant of GOAT, and primers GOAT Forward and Reverse V2 were used for the second variant). Genes were amplified using 1 μl of cDNA and 0.5 μM of each forward and reverse primer in a final volume of 10 μl. Each PCR run included water controls in order to ensure that the reagents were not contaminated. The qPCR cycling conditions consisted of a ramp of 95°C for 30 sec and 40 cycles of a two-step amplification program (95°C for 5 sec and 60°C for 30 sec). A melting curve was systematically monitored (temperature gradient at 0.5°C/5 sec from 65 to 95°C) at the end of each run to confirm the specificity of the amplification reaction. The PCR products were checked by electrophoresis on a 1.5% agarose gel. The 2-ΔΔCt method [37] was used to determine the relative mRNA expression, considering the tissue with the lowest expression levels as the relative value of ‘1’.

### Immunohistochemical analysis of GOAT and ghrelin cellular localization within the intestine

The intestine (including esophagus, intestinal bulb and j-loop) from 72-h food deprived goldfish was collected in 4% paraformaldehyde (PFA) for 24 h. Tissues were processed (dehydrated and embedded in paraffin) and sectioned (5 μm thickness) at the Centre for Modeling Human Disease (Toronto, Canada). Sections were deparaffinized in xylene for 10 minutes, rehydrated in increasing concentrations of ethanol, dried and treated with 3% hydrogen peroxide for 30 min at room temperature. Slides were then washed for 10 min in a solution of phosphate buffered saline (PBS) and Kodak Photo-Flo, followed by 10 min in PBS and Triton X-100, and 10 min in PBS and antibody blocking solution. Then, slides were incubated with a mixture of 1:200 primary ghrelin antibody (mouse monoclonal anti-ghrelin; catalog # ab57222; Abcam, Toronto, Canada) and 1:200 GOAT antibody (Rabbit monoclonal anti-GOAT; catalog # H-032-12; Phoenix Pharmaceuticals, Burlingame, USA) during 24 h. Both primary antibodies were previously validated in zebrafish [22]. The GOAT antibody targets a part of the GOAT sequence located close to the C-terminal extreme, which appears in both of the GOAT isoforms described by us. A separate set of control slides were only treated with the antibody diluent. The following day, the slides were washed and subsequently incubated in secondary antibodies for 60 minutes at 37°C. A dilution 1:200 of FITC Green was used for ghrelin and 1:300 was used for Texas-Red that bound to the GOAT primary antibody. Slides were then washed as follows: 10 min in a solution of 90 mL PBS and 600 μl Kodak Photo-Flo, 10 min in 90 mL PBS and 1 drop Triton X-100, 10 min in 90 mL PBS and 600 μl Kodak Photo-Flo, 10 min in 90 mL distilled water and 600uL Kodak Photo-Flo (three times), and 10 min in distilled water (three times). Finally, slides were mounted using Vectashield^®^ mounting medium containing the DAPI nuclear fluorescent dye (Vector Laboratories, Burlington, Canada). Images of epithelial cells lining the villi were taken using a Nikon Eclipse Ti-Inverted fluorescent microscope (Nikon Canada, Mississauga, Canada) and NIS-elements imaging software (Nikon Canada).

### *In vitro* study of the possible modulatory role of ghrelin on GOAT gene and protein levels

An explant culture was performed as previously described [38] with slight modifications. Briefly, the anterior intestine (4–5 cm after the intestinal bulb) of 24 h-fasted goldfish (n = 6) was removed and cut into fragments of approximately 1–2 mm width. Intestinal portions were immersed in Dulbecco’s Modified Eagle Medium (DMEM; Thermo Fisher Scientific, New York, USA) supplemented with 44 mM sodium bicarbonate, 10% penicillin-streptomycin and 0.5% gentamicin for 1 min, and then distributed in different wells of sterile culture 24-well multidish plates. Plates were preincubated for 2 h in 1 ml of DMEM supplemented with 44 mM sodium bicarbonate, 1% penicillin-streptomycin and 0.05% gentamicin (DMEM+) at 23°C under an atmosphere of 5% CO_2_ and 95% O_2_. Then, medium was replaced by 1 ml of fresh DMEM+ alone or containing goldfish acylated ghrelin (0.1, 1 or 10 nM; GTS(Octanoyl)FLSPAQKPQGRRPPRM; GenScript, Scotch Plains, USA), and plates were incubated for 30, 60 or 120 min. At the end of each culture time, intestine samples were collected, quickly frozen in liquid nitrogen and maintained at -80°C until analytic determinations were performed. For quantifying *goat* mRNA levels, total RNA extraction, cDNA synthesis of 1 μg of total RNA and qPCRs were carried out as described above. Primers used for qPCRs of *goat* (GOAT Forward 4 and Reverse 5; Table 1) were able to amplify both of the described variants of the enzyme. *Ef-1α* and *β-actin* were used as reference genes.

For quantifying protein levels, tissues (n = 3) were homogenized in T-PER tissue protein extraction reagent (Thermo Fisher Scientific) followed by measurement of protein concentration by Bradford assay. The samples (containing 30 μg protein) were prepared in 1x Laemmli buffer containing 0.2% 2-mercaptoethanol (Bio-Rad, Mississauga, Canada) and subsequently were boiled at 95°C for 10 min. Then, the whole sample volume was loaded and electrophoresed in a 8–16% Mini-PROTEAN^®^ TGX^™^ precast protein gel (Bio-Rad Canada). Precision plus protein^™^ Dual Xtra standards (Bio-Rad Canada) was used as molecular weight marker. Following electrophoresis, proteins were transferred to a 0.2 mm BioTrace^™^ nitrocellulose membrane (Bio-Rad Canada) using the Trans-Blot^®^ Turbo^™^ transfer system (Bio-Rad, Canada), and membrane was blocked in 1x RapidBlock^™^ solution (aMReSCO, Toronto, Canada). Total GOAT protein detection was performed using rabbit polyclonal IgG to GOAT (1:5000 dilution; catalog # sc-87999; Santa Cruz Biotechnology, Dallas, USA) primary antibody. Vinculin protein was used for normalization and was detected using rabbit antiserum directed against mouse vinculin (1:2000 dilution; catalog # ab129002, Abcam). As secondary antibody, goat anti-rabbit IgG (H+L) HRP conjugate (Bio-Rad Canada) diluted 1:2000 was used. For protein visualization the membrane was incubated for 5 min in Clarity^™^ Western ECL substrate (Bio-Rad Canada) and imaged using ChemiDoc^™^ MP imaging system (Bio-Rad Canada) with chemiluminescence detection. Blot images were plotted using ImageJ software and band density of vinculin was used to normalize GOAT protein density.

### Influence of feeding time on 24-h *goat* mRNA expression

Fish maintained under a 12L:12D photoperiod were divided into two groups: one fed during the photophase (at ZT-6, midday) and the other during the scotophase (at ZT-18, midnight) with commercial flake diet during three weeks. The locomotor activity was recorded as a circadian output to ensure that fish were entrained to the different feeding conditions. For this, infrared photocells (OMRON E3S-AD12, Japan) were fixed on different parts of the aquaria walls. These cells continuously emitted an infrared light beam, which was interrupted each time a fish swam in that zone, generating an output signal. The number of light beam interruptions was automatically counted and stored every 10 min by specific software (Micronec, Spain). The experiment was carried out once fish were entrained to the different feeding conditions. The day of the experiment, fish were sacrificed in 4 h intervals throughout a complete 24-h cycle beginning at *zeitgeber* time 5 (ZT-5, 6 fish/sampling time). Food was offered as scheduled the day of the experiment. Once sacrificed, samples of telencephalon, hypothalamus, vagal lobe, pituitary and intestinal bulb were collected and immediately frozen in liquid nitrogen. Sampling during darkness was conducted under dim red lighting. Total RNA extraction, cDNA synthesis of 0.3 μg of total RNA and qPCRs were carried out as described above. Primers used for quantifying *goat* (GOAT Forward 4 and Reverse 5; Table 1) were able to amplify both of the described variants of the enzyme. *Ef-1α* and *β-actin* were used as reference genes. For the determination of the relative mRNA expression using the 2-ΔΔCt method [37], the relative value of ‘1’ was assigned to the sampling time with the lowest expression values.

### Statistics

Statistical differences in GOAT mRNA or protein expression among different tissues (for the tissue distribution study), different experimental groups (for the *in vitro* assay) and different time points (for the circadian study) were assessed by one-way ANOVA followed by post-hoc Student-Newman-Keuls multiple comparison test, after data were checked for normality (Shapiro-Wilk test) and homogeneity of variance (Levene’s test). Data that failed one of these requirements were log-transformed and re-checked. Analyses were carried out using SigmaStat 12.0 statistics package and significance was assigned at p<0.05. In addition, the rhythmicity of gene expression for Section 2.7 was assessed by cosinor analysis, by fitting periodic sinusoidal functions to the expression values for the genes across the six time points. The formula used was f(t) = M+Acos(tπ/12−ϕ), where f(t) is the gene expression level in a given time, the mesor (M) is the mean value, A is the sinusoidal amplitude of oscillation, t is time in hours and ϕ is the acrophase (time of peak expression). Non-linear regression allows the estimation of M, A, and ϕ, and their standard error (SE) [39]. Significance of cosinor analysis was tested using the zero-amplitude test, which indicates if the sinusoidal amplitude differs from 0 with a given probability [40]. Circadian variations were considered to be significant when p<0.05 by ANOVA, and p<0.005 by the zero-amplitude test with cosinor analysis.

## Results and discussion

### Two variants of GOAT are identified in goldfish

**Fig 1:**
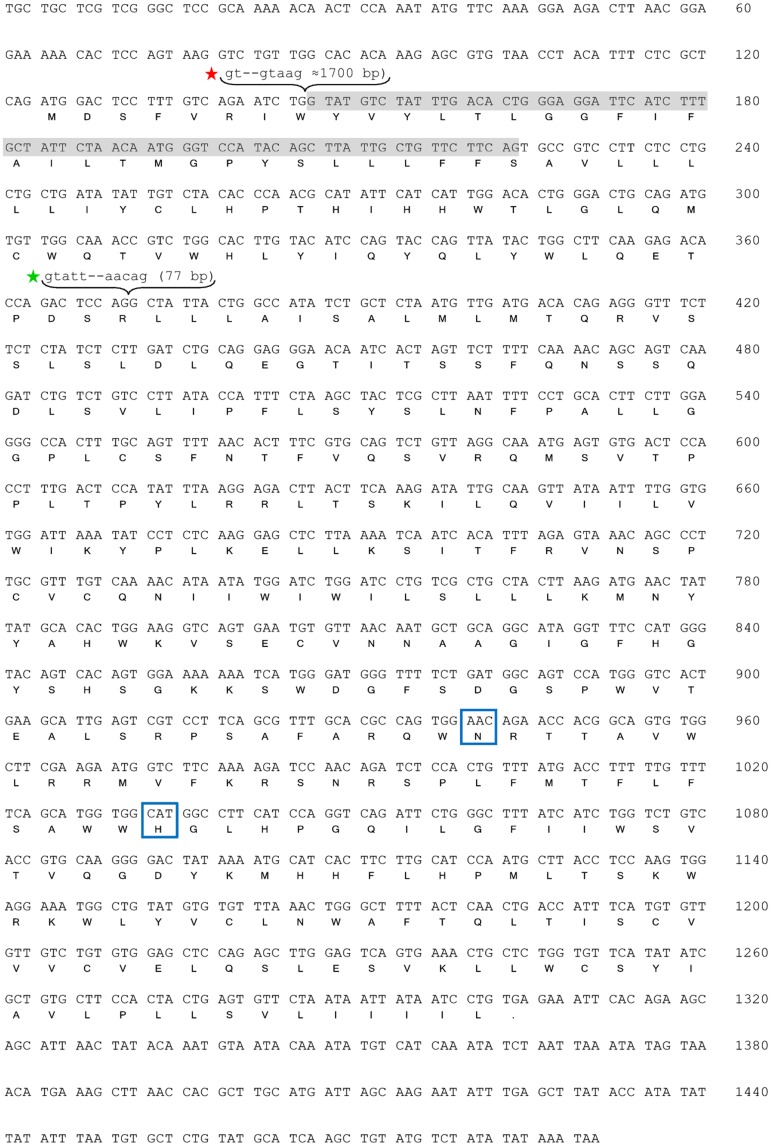
Nucleotide and deduced amino acid sequences of goldfish GOAT. The full-length sequence corresponds to GOAT-V1, and the same sequence without the shaded nucleotides and amino acids represents GOAT-V2. Both sequences are accessible through GenBank (accession numbers KX953158 and KX953159). Encoding region of sequences extends from the first methionine residue to the stop codon (asterisk, *). The red star indicates the union point between the first exon (exon 1’) and exon 2. The sequence between these two exons comprises two introns (1’ and 1) separated by exon 1. The green star indicates the intron between the exons 2 and 3. The 5’ and 3’ extremities of these intronic sequences are indicated in lowercase letters, and their size is indicated between brackets. The proposed catalytic residues of GOAT (asparagine 273 and histidine 304) are boxed.

**Fig 2:**
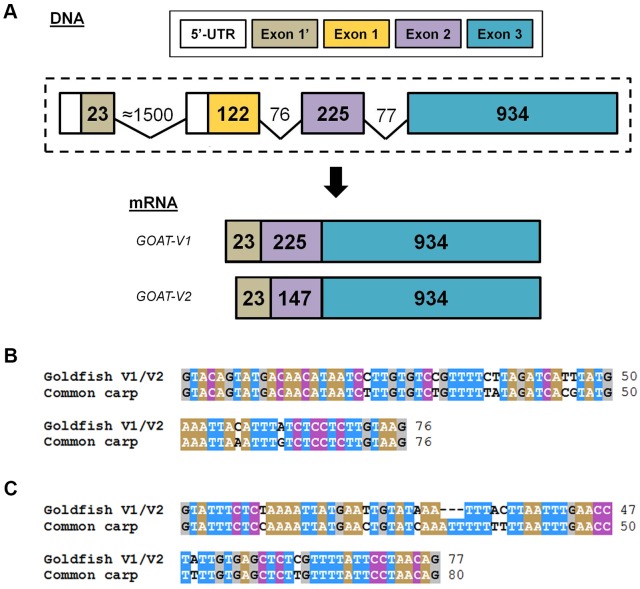
Exon:intron structure and proposed transcription pattern of the *goat* gene in goldfish. (A) Schematic illustration representing the proposed model for the exon-intron structure of the gene encoding GOAT in goldfish and its pattern of transcription into mRNA. Exons are indicated by boxes and introns by lines. The length (pb) of exons and introns is indicated inside the boxes (exons) or above lines (introns). (B—C) Alignment of the nucleotide sequences of the intron 1 (between exons 1 and 2, B) and intron 2 (between exons 2 and 3, C) of GOAT-V1 and GOAT-V2 DNAs with the corresponding intron sequences inferred from the common carp genome. Multiple sequence alignments were conducted using Clustal W2 (http://www.ebi.ac.uk/Tools/msa/clustalw2/) and edited using the BioEdit Sequence Alignment Editor. Identical amino acids between the two sequences are colored. The common name of the species used for the alignment is given on the right side, and the species name and GenBank accession number of the common carp is as follows: *Cyprinus carpio*, LHQP01003245.1(80257–80106….78407–78390).

The identification of GOAT full-length sequence from goldfish tissues allowed us to distinguish the presence of two transcripts of 1491 and 1413 bp, respectively, named goldfish *goat-V1* and *goat-V2* (GenBank accession numbers KX953158 and KX953159, respectively). The nucleotide sequences of both variants are identical, except for 78 nucleotides (located between positions 23 and 101) that are present in *goat-V1* and missing in *goat-V2* (Fig 1). The structure of the gene encoding GOAT is conserved along the phylogeny, from fish to mammals, and responds to a 3 exons and 2 introns-gene [19]. Our results from PCRs using genomic DNA demonstrated that the goldfish *goat* has such typical 3 exons:2 introns structure, but, contrary to what expected, the first exon in goldfish is not homologous to the first exon of *goat* in other vertebrates. An *in silico* analysis of the *goat* sequences of other members of the Cyprinidae family (*Danio rerio*, *Sinocyclocheilus sp*. -horned golden-line barbel- and *Cyprinus carpio*) has revealed the presence of this different exon 1 (referred to as exon 1’) in their genome, with an extremely high degree of identity in all the species (S1 and S2 Figs). Both this exon 1’ and the typical exon 1 are present in the genome of zebrafish, horned golden-line barbel, and common carp, indicating that the *goat* gene in these fish species would actually contain four exonic sequences (S1 Fig). The typical exon 1 is also present in the goldfish genome, as revealed by present results (S1 and S3 Figs). Thus, the goldfish *goat* gene would also be composed of four exons (1’, 1, 2 and 3) of 23, 122, 225 and 934 bp, respectively, separated each other by intronic sequences (Fig 2A and S1 Fig). The intron 1’ (between exons 1’ and 1), based on homology among species, seems to have around 1500 bp, while the introns 1 (between exons 1 and 2) and 2 (between exons 2 and 3) are shorter and are formed by 76 and 77 bp, respectively (Fig 2A and S1 Fig). These two introns are identical in both goldfish *goat* forms (V1 and V2), and their nucleotide sequences are shown in Fig 2B and 2C, respectively. Alignment of these sequences with the deduced intronic nucleotide sequences of the common carp revealed an extremely high percentage of similarity between the two species for the two introns.

It must be highlighted that either the exon 1’ or the exon 1 are transcribed into mRNA. That means that if transcription begins in exon 1, the resulting mRNA is composed of transcripts from exon 1, exon 2 and exon 3, which is the case of the typical *goat* found in most vertebrates. However, experimental evidences presented here point out that the exon 1’ is to be transcribed in the goldfish, so resulting in a mRNA composed of transcripts from exon 1’, exon 2 and exon 3. If exon 2 is fully transcribed, that would result in *goat-V1*, while *goat-V2* would be generated by alternative 3’ splice site selection at this exon (Fig 2A and S1 Fig). Furthermore, two characteristics can be highlighted from the 78 nucleotide-fragment missing in *goat-V2*: i) it begins with the nucleotides GT and ends with the nucleotides AG (typical extremities of intronic sequences), and ii) its number of nucleotides is multiple of three, and so the reading frame of the protein is not altered by frame shifting. Given these facts, it can be plausible that the intron elimination occurs in sequential steps, i.e. first resulting in the *goat-V1* mRNA, and then in *goat-V2* mRNA. In addition, it is plausible that a second gene encoding *goat* might exist in goldfish, as it is present in other members of the Cyprininae subfamily, whose members underwent an additional whole genome duplication (4R) during evolution [41]. This second gene appears to be functional in species from the Barbini tribe (such as the horned golden-line barbel), but not in the Cyprinini tribe (such as the common carp). The additional intron inserted within the third exon, the four nucleotides deletions that lead to a shift in the codon reading frame, and the presence of a premature stop codon, support the functional loss of this second *goat* gene in common carp (S1 Fig). Therefore, it is likely that goldfish, as another member of the Cyprinini tribe, also contains a second gene encoding GOAT that might have been pseudogenized.

**Fig 3:**
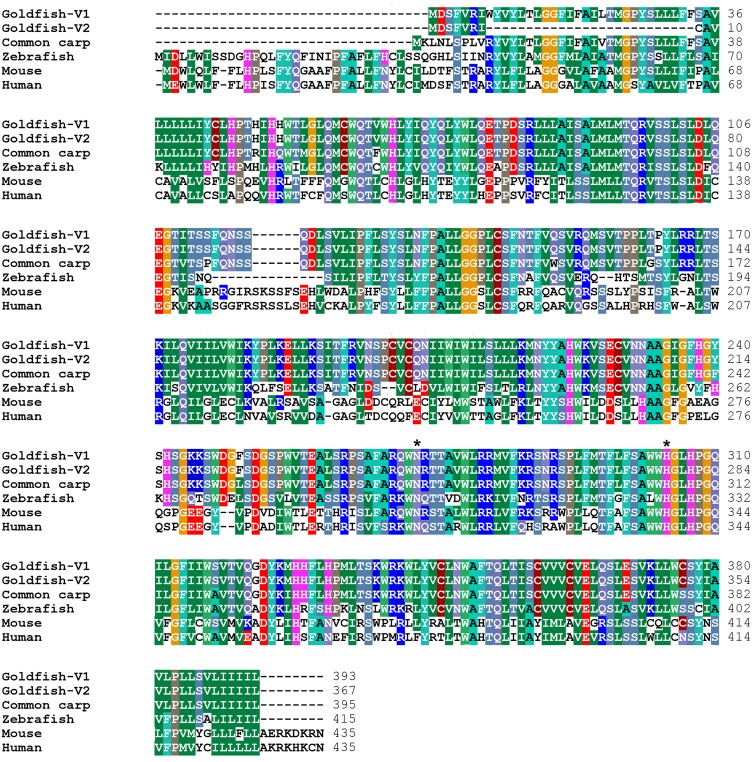
Alignment of the deduced amino acid sequences from goldfish GOAT-V1 and GOAT-V2 with GOAT from common carp, zebrafish, mouse and human. Multiple sequence alignment was conducted using Clustal W2 (http://www.ebi.ac.uk/Tools/msa/clustalw2/) and edited using the BioEdit Sequence Alignment Editor. Dashed lines represent voids introduced to optimize the alignment. Identical amino acids among sequences are colored, and the proposed catalytic residues of GOAT (asparagine and histidine) are marked by asterisks. The common name of the species used for the alignment is given on the right side, and the species names and GenBank accession numbers are as follows: common carp, *Cyprinus carpio*, LHQP01003245.1(80257–80106….78407–78390); human, *Homo sapiens*, ACB05873.2; mouse, *Mus musculus*, ACB05874.1; zebrafish, *Danio rerio*, CABZ01049031.1(6992–7150…18211–18228).

The open reading frame of both goldfish *goat-V1* and *goat-V2* has the potential to code for a 393 and a 367 amino acid proteins, respectively. Molecular weight of GOAT-V1 and GOAT-V2 is 45.562 kDa and 42.549 kDa, respectively, and they both contain 28 positively and 14 negatively charged amino acids. It is worthy to highlight that the start codon (methionine) that initiates the translation of GOAT in many vertebrates is absent in goldfish because of the different first exon of its gene. Therefore, there might be another start codon determining the synthesis of both goldfish GOAT-V1 and GOAT-V2. In this respect, it has been reported that the methionine in position 56 is an alternate start codon in mouse GOAT [23], resulting in a shorter GOAT compared to the typical one. This methionine is present in GOAT-V1, but not in GOAT-V2; then, it is highly likely that at least the goldfish GOAT-V1 exhibits a shorter form that might be functional. However, both GOAT-V1 and GOAT-V2 have a previous methionine that could act as the start codon in goldfish GOATs, leading to functional full-lengths proteins. The alignment of the deduced amino acid sequences from the two variants of goldfish GOAT with the deduced amino acid sequences from common carp and zebrafish shows a high grade of similarity. There is also an important number of coincident amino acids with the mouse and human sequences, particularly in the regions of the protein that are encoded by the second and third exons of the genes (Fig 3). Indeed, the active core of GOAT is encoded by the last exon and it contains the two residues (asparagine and histidine) considered essential for the catalytic activity of most members of the MBOAT family, including GOAT. These residues are present in both goldfish GOAT-V1 and GOAT-V2 sequences (Figs 1 and 3).

**Fig 4:**
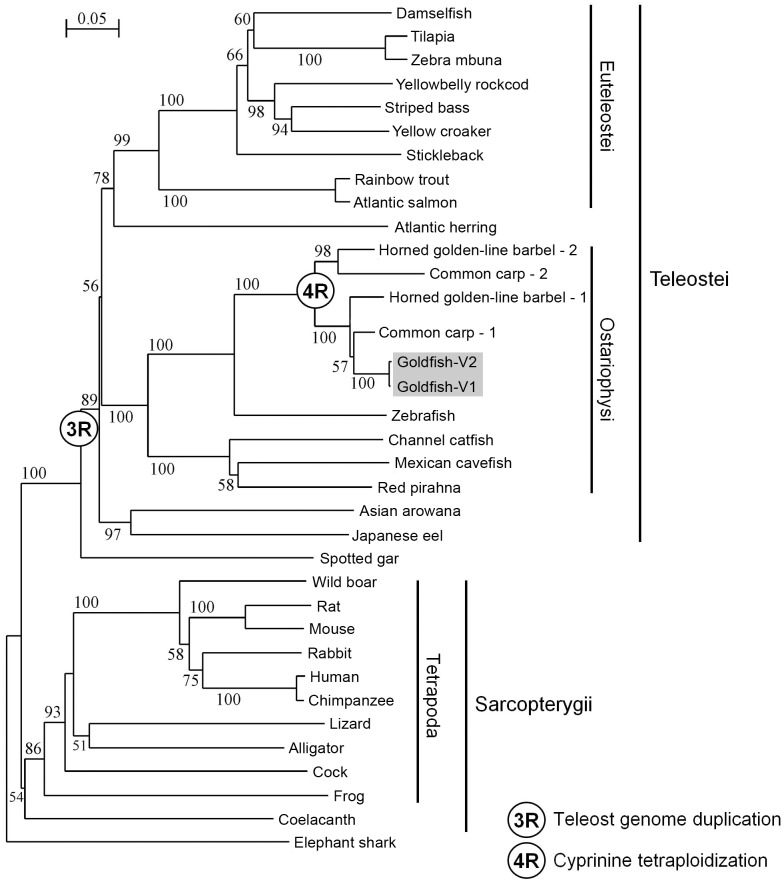
Phylogenetic analysis of GOAT sequences. A phylogenetic tree showing the evolutionary relationships of the deduced amino acid sequences of goldfish GOAT with those of other species was inferred by the neighbor-joining method using MEGA6 [36]. The numbers at tree nodes refer to percentage of trees in which the associated taxa clustered together in the bootstrap test (1000 replicates). The scale bar indicates the average number of substitutions per position (a relative measure of evolutionary distance). The common name of the species used for the alignment is given on the right side of the tree, among which goldfish is shaded. Species names and GenBank and Ensembl accession numbers of the sequences used are as follows: alligator, *Alligator sinensis*, XP_006035341.1; Asian arowana, *Scleropages formosus*, JARO02002481.1(36315–36436….36746–36978….37427–38368); Atlantic herring, *Clupea harengus*, JZKK01021833.1(58124–58006….52933–52709….50211–49272); Atlantic salmon, *Salmo salar*, XP_014016526.1; channel catfish, *Ictalurus punctatus*, XP_017306886.1; chimpanzee, *Pan troglodytes*, ENSPTRT00000037288; cock, *Gallus gallus*, NP_001186218.1; coelacanth, *Latimeria chalumnae*, BK009986; common carp, *Cyprinus carpio*, (1) LHQP01003245.1(78599–78478…78401–78176…78096–77163) and (2) LHQP01015814.1(64651–64772…64875–65084…65162–65417…67215–67900); damselfish, *Stegastes partitus*, XP_008292386.1; elephant shark, *Callorhinchus milii*, BK009985; frog, *Xenopus tropicalis*, XP_002936505.2; goldfish, *Carassius auratus*, (V1) APD26025 and (V2) APD26026; horned golden-line barbel, *Sinocyclocheilus rhinocerous*, (1) XP_016428796.1 and (2) XP_016383356.1; human, *Homo sapiens*, ACB05873.2; Japanese eel, *Anguilla japonica*, AVPY01018663.1(5671–5450…5379–5155…4515–3518); lizard, *Anolis carolinensis*, XP_003224702.1; Mexican cavefish, *Astyanax mexicanus*, XP_007253942.1; mouse, *Mus musculus*, ACB05874.1; rabbit, *Oryctolagus cuniculus*, ENSOCUT00000014851; rainbow trout, *Oncorhynchus mykiss*, CDQ71181.1; rat, *Rattus norvegicus*, ACB05875.1; red pirahna, *Pygocentrus nattereri*, MAUM01004312.1(861–985…1063–1287…2692–3625); spotted gar, *Lepisosteus oculatus*, BK009987; stickleback, *Gasterosteus aculeatus*, AANH01001771.1(95917–95796….95445–95215….95100–94149); striped bass, *Morone saxatilis*, JTCL01001059.1(43688–43809….44568–44792….45768–46719); tilapia, *Oreochromis niloticus*, AERX01036891.2(7728–7607….6577–6353….4935–3987); wild boar, *Sus scrofa*, ADI55170.1; yellow croacker, *Larimichthys crocea*, XP_010729215.1; yellowbelly rockcod, *Notothenia coriiceps*, AZAD01045919.1(5788–5667….5115–4891….4240–3295); zebra mbuna, *Maylandia zebra*, XP_014262684.1; zebrafish, *Danio rerio*, ACB05876.1. Nucleotide sequences were translated to amino acids using Wise2 (http://www.ebi.ac.uk/Tools/psa/genewise/).

Phylogenetic analysis comparing the goldfish GOAT amino acid sequences and those from other vertebrates positions the elephant shark GOAT in the base of the evolutionary scale, in agreement with the concept that cartilaginous fish originated before the rest of the vertebrates. The phylogenetic tree shows the existence of two clear clades that separate bony fish from tetrapodian GOAT, except for the coelacanth, an extant species of Sarcopterygii which is actually more closely related to lungfish, reptiles and mammals than to the common ray-finned fishes (Fig 4 and S4 Fig). It must be noted that the second copy of the *goat* gene of all teleosts, as a consequence of the whole genome duplication that this group of fish underwent (3R), seems to have been lost during evolution, as only one copy is found in all the species studied. Only members from the Cyprininae subfamily, which underwent an additional genome duplication (4R), have a second copy of the gene. The goldfish GOAT sequences here identified appear closely related to sequences from other cyprinids (common carp, horned golden-line barbel and zebrafish), with a strong bootstrap support (100%). Specifically, the amino acid sequence identity for goldfish GOAT compared to common carp and zebrafish was 96% and 70%, respectively. The highest sequence divergence of goldfish GOAT was observed with cock and human, sharing 39% and 38% amino acid sequence identity, respectively. These results support that a considerable grade of conservation exists among GOAT amino acid sequences across vertebrates. This sequence conservation seems to be translated also into functional terms, since the mouse, rat, and zebrafish forms of GOAT are able to acylate human ghrelin [19].

**Fig 5:**
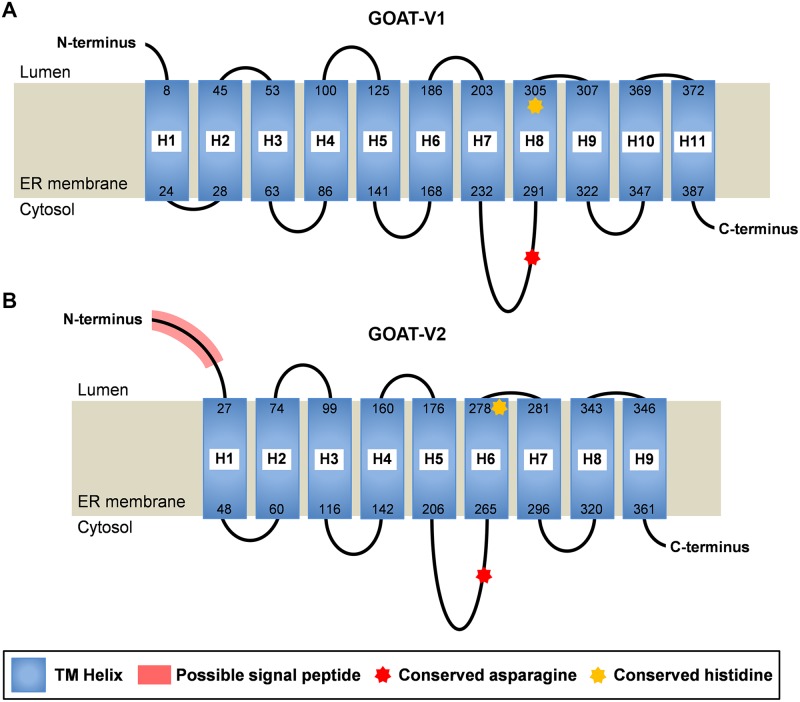
Predicted topological organization of goldfish GOAT-V1 (A) and GOAT-V2 (B) in the membrane of the endoplasmic reticulum. Topology was predicted using MemBrain prediction server [42,43]. Transmembrane helices are represented by blue rectangles spanning the endoplasmic reticulum membrane (gray). Black lines represent loops, and their size indicates the relative loop length (although they are not precisely scaled). GOAT-V2 is predicted to contain a peptide signal, which is shaded in the N-terminus. Positions of the two conserved catalytic residues asparagine and histidine are shown with red and yellow stars, respectively.

An *in silico* study was carried out to predict the topology of the newly identified variants of goldfish GOAT within the membrane of the endoplasmic reticulum. For this, a battery of web-based topology prediction servers were tested, including DAS, MemBrain, MEMSAT-SVM, Phobius, PredictProtein, Protter, SOSUI, TMHMM, TMPred and TopPred 1.10. Most servers gave very different results and predicted a very different number of TMDs; the range was from 5 to 11 for GOAT-V1 and from 5 to 9 for GOAT-V2. Among the different servers tested, MemBrain [42,43] and MEMSAT-SVM [44] incorporate newer machine-learning algorithms, phylogeny and PSI-BLAST strategies, and current knowledge on membrane protein structure, such as short and very long helices and reentrant loops (also referred to as half-TMs or half-helices). Thus, results on the topological organization of both variants of goldfish GOAT were obtained from predictions from these two last servers. The topological organization predicted by MemBrain is represented in Fig 5, with a total of 11 TMDs in GOAT-V1 and 9 TMDs in GOAT-V2. This server does not predict orientation for helices, but given that the C-terminus was consistently localized in the cytosol in almost all the MBOATs studied [23,45], we assumed the same for goldfish GOAT. Considering this, both GOAT-V1 and GOAT-V2 have the N-terminus located on the luminal side and the C-terminus on the cytosolic side of the endoplasmic reticulum. This orientation of both extremities is in accordance with the predicted topology of GOAT in some vertebrates, including many species of mammals, chicken, green anole, frog and zebrafish [23]. MEMSAT-SVM predictions were very similar to those from MemBrain, with two differences. First, according to this model, the amino acids predicted by MemBrain to form the TMD5 of GOAT-V1 do not constitute a transmembrane helix but they are part of the corresponding cytosolic loop. This means a total number of 10 TMDs, and the positioning of the two extremities of the protein in the cytosolic side of the endoplasmic reticulum membrane. Second, the TMD6 and TMD7 predicted by MemBrain for GOAT V2 are predicted as a unique longer TMD by MEMSAT-SVM, with a reduction of TMDs from 9 to 8, and the cytosolic location of both the N- and C-terminus of the protein. As the goldfish *goat* has a shorter first intron compared to other vertebrates and, therefore, a shorter GOAT protein, its architecture resembles the one previously described for many vertebrates [23], which comprises 11 TMDs and one reentrant loop.

According to MemBrain and MEMSAT-SVM predictions, in both variants of goldfish GOAT the conserved asparagine residue is part of a long cytosolic loop, while the conserved histidine is segregated to the luminal side of the endoplasmic reticulum membrane (Fig 5). This location of these two key residues is in accordance with the previous predicted model for GOAT topology [23]. The luminal location of histidine is in consistence with the hypothesis of a conserved luminal active site for the MBOAT family. In contrast, the cytosolic position of the asparagine suggests that it is unlikely to be directly involved in catalysis, although it might be important for substrate interactions, transport of substrates or protein structural stability, among others. Another important finding is the prediction of the existence of a peptide signal within the goldfish GOAT sequences (Fig 5). None of the prediction tools used detected a signal peptide for GOAT-V1, in agreement with the results obtained by Taylor and coworkers [23]. However, results were not so consistent for GOAT-V2. While some servers, such as SignalP4.1, MemBrain, and Protter and Phobius, predicted that the first 24 amino acids (18 according to MemBrain) of the sequence constitute a signal peptide, MEMSAT-SVM failed to detect one. This difference regarding the presence of a signal peptide in the GOAT-V2, but not in the GOAT-V1, could indicate a different subcellular location of both forms of goldfish GOAT. Further studies are required to demonstrate this hypothesis.

Apart from GOAT [23], three other MBOATs have been fully mapped topologically: the human acyl-CoA:cholesterol acyltransferases 1 and 2 (ACAT1 and ACAT2) [46] and yeast glycerol uptake protein 1 (Gup1p) [45]. The topological models described for ACAT1 and Gup1p are similar to the one reported for GOAT, whereas ACAT2 shows a quite different organization. Although the number of TMDs predicted differs among these MBOATs, GOAT, ACAT1 and Gup1p have in common that the invariant histidine is luminal or buried in the endoplasmic reticulum membrane near the lumen, as reported in the present study for goldfish GOATs (Fig 5). This was also reported for the partially mapped yeast MBOATs acyltransferase for lysophosphatidylethanolamine 1 (Ale1p) and acyl-CoA:cholesterol acyltransferase-related enzymes 1 and 2 (Are1p and Are2p) [45]. Furthermore, it is to note that this histidine is in all cases part of or very close to a cytosolic loop containing only 2–3 residues, a feature that is conserved throughout the MBOAT gene family [45]. Regarding the conserved asparagine, all these previous studies reported its position as cytosolic, in accordance to our model.

### The two variants of goldfish *goat* show a similar tissue expression pattern

**Fig 6:**
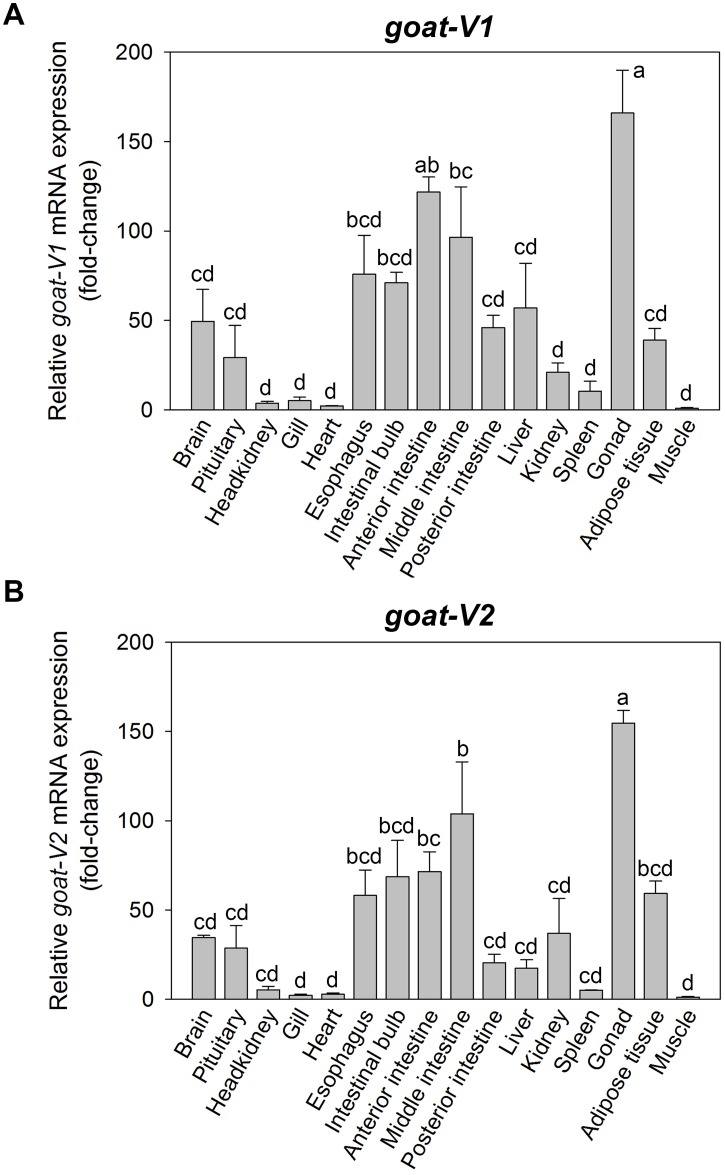
Tissue distribution of *goat-V1* (A) and *goat-V2* (B) mRNAs in goldfish. Quantitative analysis of mRNA expression was performed by RT-qPCR considering *elongation factor-1α* (*ef-1α*) as reference gene. Data are expressed as mean + SEM (n = 4), relative to the tissue with the lowest mRNA expression. Bars not showing any coincident letter indicate that their difference in mean is statistically significant, as assessed by ANOVA and post-hoc SNK test (p<0.05).

The distribution and expression of goldfish *goat-V1* and *goat-V2* transcripts in brain and peripheral tissues of goldfish are shown in Fig 6A and 6B, respectively. Both variants of the enzyme show a similar tissue expression pattern, with the highest levels of mRNAs detected in gonads followed by the anterior intestinal tract, including the esophagus, intestinal bulb, anterior intestine and middle intestine. Lesser but significant levels of *goat* expression were found in the brain, pituitary, posterior intestine, liver, kidney and adipose tissue. It is to note that the expression of *goat-V1* in the posterior intestine and liver is higher than *goat-V2*, which is more expressed in head kidney in comparison to *goat-V1*. Expression of both forms was found to be minimal in head kidney, gill, heart and spleen, and almost undetectable in muscle.

The tissue expression pattern observed for goldfish *goat* is similar to the reported previously in other vertebrates (zebrafish, [22]; mammals, [19,20]), with high levels of *goat* mRNAs in gut and gonads. The broadly *goat* expression in the intestinal bulb and intestine in goldfish supports the functional activity of the ghrelinergic system in such locations and its relationship with feeding [35,47]. On the other hand, the high presence of *goat* transcripts in gonads supports the previously suggested involvement of ghrelin in reproduction [48]. Besides, a significant presence of *goat* has been detected in the pituitary, which may be related to the important role of ghrelin in stimulating hormone secretion (including growth hormone and luteinizing hormone) in this gland [49]. While the two forms of *goat* here identified (V1 and V2) are similarly expressed in the above mentioned locations, and so they might be equally involved in the suggested functions, their expression is quite different in the posterior intestine, liver and head kidney. This observation points out that GOAT-V1 might be involved in the actions of ghrelin in the posterior intestine and liver, while actions exerted by the ghrelinergic system in the head kidney might be carried out by GOAT-V2.

It is worthy to highlight that none of the studies analyzing the tissue expression pattern of *goat* in mammals detected mRNAs encoding the enzyme in the brain [19,20], in contrast to the significant levels found in goldfish (present results) and in zebrafish [22]. However, a significant amount of *goat* mRNAs has been detected in the rat hypothalamus [32]. These results could indicate that GOAT in mammals might be restrictedly distributed in discrete brain areas (such as the hypothalamus), and then is detected only when such discrete locations are analyzed separately, while in fish it is more widely expressed within this tissue [50]. The important presence of *goat* in the goldfish brain and gastrointestinal tract reinforces the crosstalk between these two tissues in the regulation of food intake [51].

### GOAT is present in the goldfish intestinal mucosa and colocalizes ghrelin in some endocrine cells

**Fig 7:**
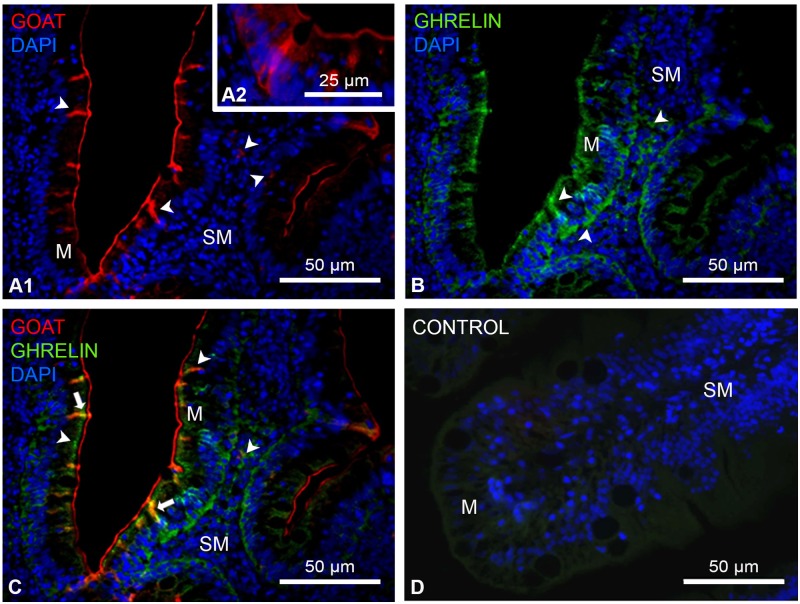
GOAT-like and ghrelin-like immunoreactivity in goldfish intestine detected by immunohistochemistry. Figure shows transversal representative sections of intestine showing GOAT-like (A1 and A2, red) and ghrelin-like (B, green) immunoreactivity, and merged images of GOAT and ghrelin (C, yellow). Slides labeled only with secondary antibodies were used as negative controls (D). Arrowheads indicate cells stained with either GOAT or ghrelin, and solid arrows show cells that colocalize both GOAT and ghrelin. All images are merged with DAPI showing nuclei in blue. M, mucosa; SM, submucosa. Scale bars are indicated in each image.

Our findings indicate that *goat* transcripts are considerably expressed along the intestine. Then, we aimed to characterize the anatomical distribution of GOAT in the intestinal endocrine cells and whether it colocalizes ghrelin. Cross-sections of goldfish intestine stained for GOAT-like and ghrelin-like immunoreactivity are shown in Fig 7. Immunoreactivity for GOAT (red) was mainly observed within the apical region of the cells of the intestinal mucosa, and also some immunopositive cells were scattered deep within the submucosa of the villi (Fig 7A1 and 7A2). Ghrelin immunoreactivity (green) was detected both in the brush border and the basal border of the mucosa, and in the submucosa (Fig 7B). There was a small population of enteroendocrine cells colocalizing GOAT and ghrelin in the apical region of the mucosa (Fig 7C, yellow). No staining was observed in negative controls stained with secondary antibody alone, indicating that the staining is specific (Fig 7D).

The identification of GOAT immunopositive cells in the intestine of goldfish, as well as the colocalization of the enzyme with its main substrate ghelin, is in agreement with previous observations in mammals [28,29] and with the only report in fish for the zebrafish [22]. The existence of enteric endocrine cells coexpressing GOAT and ghrelin suggests that the acylation process of ghrelin is possible inside those cells, being able to produce active ghrelin when required. However, both in the present study in goldfish and the previous reports in zebrafish, rat and mice, not all the GOAT expressing cells co-express ghrelin. The percentage of co-expression of both peptides within the gastric cells varies among species. Thus, double labeling showed that around 95% of GOAT-immunoreactive cells co-labeled with ghrelin in mice [29], around 56% in rats [29], around 25% in goldfish, and only 20% in zebrafish [22]. The reason for this species differences remain to be explored, but the observation that some gastric/intestinal cells expressing GOAT do not co-express ghrelin suggests the existence of other endogenous substrate for this enzyme. In addition, it is noteworthy that GOAT was not detected in the intestinal muscular layer of goldfish, which is in accordance with previous observations reporting the absence of GHS-R1a in this layer [15]. These results, together with the fact that ghrelin seems not to modulate intestinal motility in goldfish [52], suggest that the ghrelinergic system is not exerting local effects affecting intestinal musculature in this teleost.

### Ghrelin downregulates GOAT gene and protein in goldfish intestine *in vitro*

**Fig 8:**
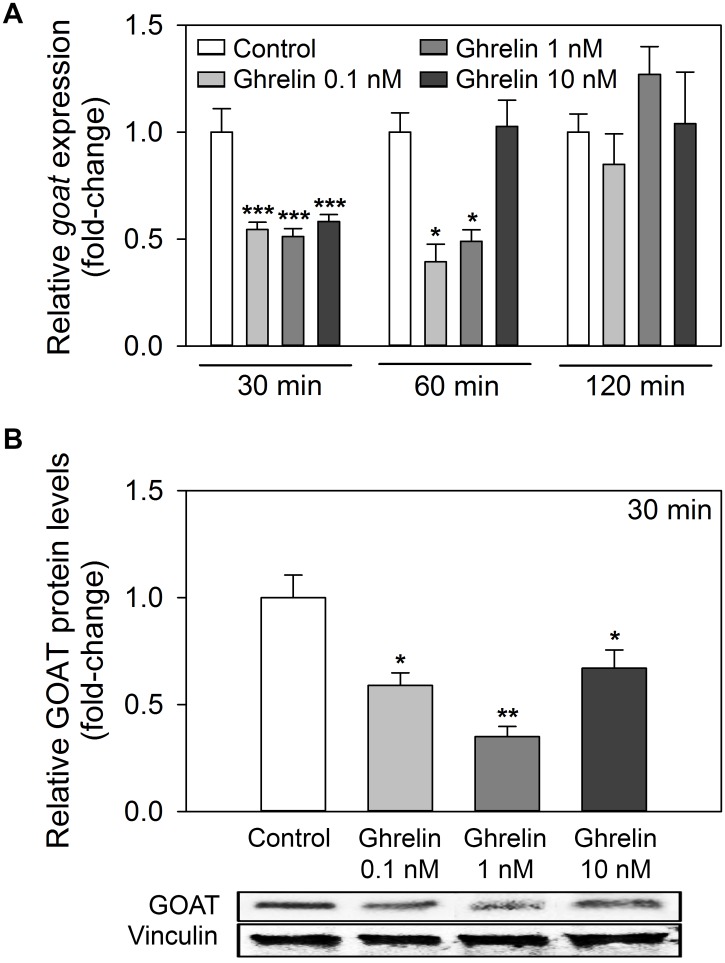
Effects of exposure of intestinal fragments to acylated ghrelin on GOAT mRNA and protein levels. (A) Concentration-response curves for *goat* mRNA expression in goldfish cultured intestine treated with goldfish acylated ghrelin (0.1, 1 and 10 nM) during 30, 60 and 120 min. Data are shown as mean + SEM (n = 6). Asterisks denote statistical differences between control and treated groups assessed by ANOVA and post-hoc SNK test (** p<0.01, *** p<0.001). (B) Concentration-response curves for GOAT protein levels in goldfish cultured intestine treated with goldfish acylated ghrelin (0.1, 1 and 10 nM) during 30 min. Bands density was quantified from three blots and normalized to the density of vinculin protein. Resulting data is plotted as mean + SEM in the upper part of the figure. Asterisks denote statistical differences between control and treated groups assessed by ANOVA and post-hoc SNK test (* p<0.05, ** p<0.01). The lower part show representative blots from one goldfish.

The effects of acylated ghrelin on the gene and protein levels of GOAT in goldfish cultured intestinal fragments are shown in Fig 8. Short time (30 min) exposure to all ghrelin concentrations tested (0.1, 1 and 10 nM) led to a significant reduction (around 2-fold) of *goat* transcript levels. This downregulatory effect on *goat* mRNA expression was also observed after 60 min incubation in the presence of lower ghrelin concentrations (0.1 and 1 nM), and disappeared at longer incubation times (120 min, Fig 8A). Incubation of intestinal fragments in the presence of ghrelin (at all the concentrations tested) also decreased significantly the intestinal levels of GOAT protein at 30 min (Fig 8B; see also S5 Fig).

Present results indicate that acylated ghrelin exerts a time-dependent inhibition of *goat* transcription and translation in the goldfish intestine *in vitro*. This observation is in agreement with the reduction of *goat* transcripts in the brain and gut induced by intraperitoneal injection of ghrelin in zebrafish [20]. However, present results show for the first time that this inhibitory role of ghrelin on GOAT occurs directly in the intestine. The existence of this mechanism is also supported by an *in vitro* assay in insect cells showing that mouse octanoylated ghrelin (1–28) and octanoylated ghrelin pentapeptide inhibit GOAT activity [53]. Together, these results points toward a possible feedback inhibition of GOAT by acylated ghrelin when an excess of this form of hormone is present in the system, highlighting the existence of a strict control on ghrelin acylation.

### *Goat* is expressed rhythmically in the hypothalamus, pituitary and intestinal bulb of goldfish fed at midday

**Fig 9:**
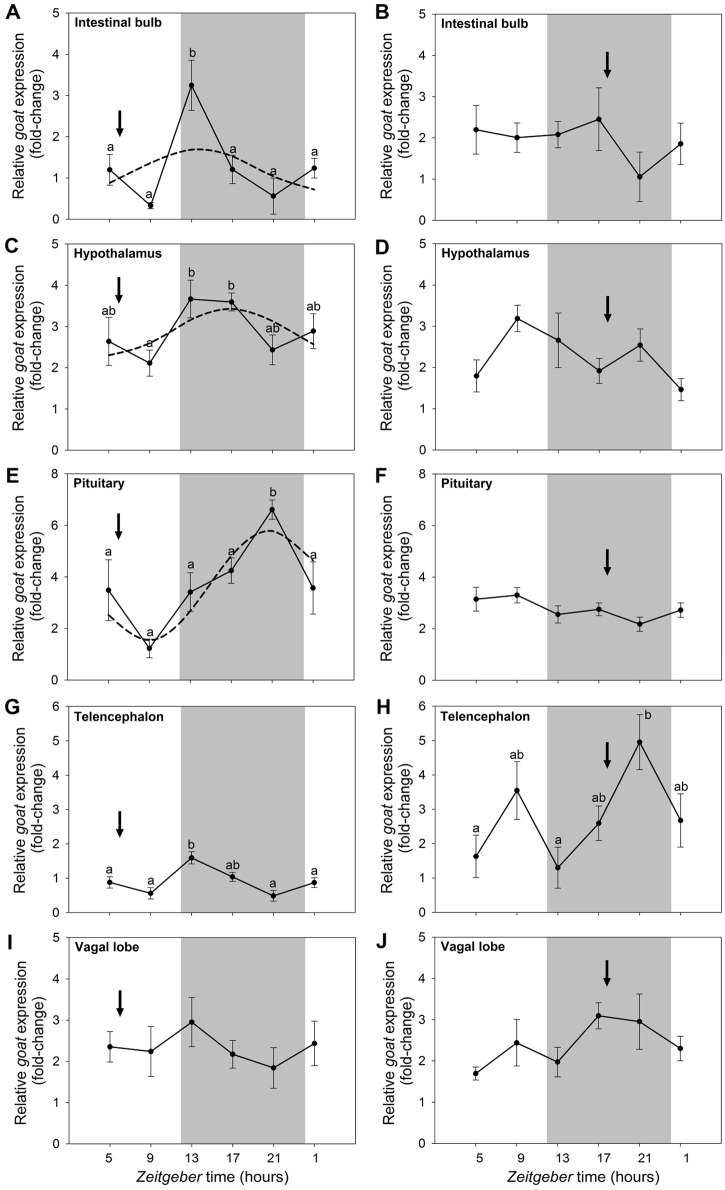
Relative expression of *goat* during a 24-h light/dark cycle in goldfish fed at midday (right panel) or at midnight (left panel). (A and B) Telencephalon, (C and D) Hypothalamus, (E and F) Vagal lobe, (G and H) Pituitary, and (I and J) Intestinal bulb. Relative mRNA amounts were quantified by RT-qPCR. Data are expressed as mean ± SEM (n = 6/time point). The grey area indicates the dark phase of the daily photocycle, and the arrow indicates the scheduled feeding time (ZT-6 or ZT-18). Dashed lines represent the periodic sinusoidal functions determined by the cosinor analysis when a significant rhythm was detected. Different letters indicate significant differences by ANOVA and post-hoc SNK test (p<0.05).

The daily pattern of *goat* expression in intestinal bulb, hypothalamus, pituitary, telencephalon and vagal lobe of goldfish fed either during the photophase or during the scotophase throughout a 12L:12D photocycle is shown in Fig 9. No differences in *goat* expression were detected throughout the 24-h cycle when fish were fed at midnight in any of the studied tissues, except in the telencephalon, where the ANOVA detected significant higher amount of *goat* transcripts at the end of the darkness compared to the midday values, but without daily significant rhythm (Fig 9B, 9D, 9F, 9H, and 9J). However, when fish were fed at midday, statistical significant differences among time points were detected in almost all the tissues (Fig 9A, 9C, 9E, 9G, and 9I), which resulted in significant daily rhythms in intestinal bulb, hypothalamus and pituitary. In these three tissues, the acrophase (time when the highest abundance of mRNAs was detected) took place during the dark phase, although in the case of the hypothalamus and intestinal bulb it was observed at the beginning of the mentioned phase whereas in pituitary it occurred at the end (Table 2). Additionally, the amplitude of *goat* daily rhythm was around 4-fold higher in pituitary compared to the hypothalamus and intestinal bulb (Table 2). Expression of *goat* was not significantly modified throughout the 24-h cycle in goldfish vagal lobe (Fig 9I and 9J). Record of the fish locomotor activity shows that both groups of fish clearly entrained their locomotor pattern to the feeding regimen (data not shown).

**Table 2:** Parameters defining the expression rhythms of *goat* in goldfish central and peripheral tissues.

	12L:12D, feeding ZT-6		
	Mesor	Amplitude	Acrophase
*Hypothalamus*	2.9 ± 0.2	0.6 ± 0.2	16.9 ± 1.8
		*[0*.*05*, *1*.*15]*	
*Pituitary*	3.7 ± 0.3	2.1 ± 0.4	20.8 ± 0.9
		*[0*.*98*, *3*.*22]*	
*Intestinal bulb*	1.2 ± 0.2	0.5 ± 0.1	13.8 ± 2.1
		*[0*.*22*, *0*.*78]*	

The confidence intervals (99%) of the amplitude values are shown in italics inside the square brackets

To the best of our knowledge, this is the first report on a daily rhythmic expression of *goat* in fishes. In a previous study, we reported daily changes in expression of *preproghrelin* and its main receptor, *ghs-r1a*, in goldfish central and peripheral tissues [15]. Present data show that the daily expression pattern of *goat* observed in hypothalamus and pituitary of goldfish fed at midday clearly matches the one reported for *preproghrelin* and *ghs-r1a* in goldfish maintained under similar conditions of photoperiod and feeding. This similar daily expression pattern in *preproghrelin*, *ghs-r1a* and *goat* points towards a coordinated function of the ghrelinergic system in such locations, and supports the importance of GOAT for ghrelin activation and functioning. In the intestinal bulb, the rhythm of *goat* transcripts is also similar to the one described for *preproghrelin* in the anterior intestine of the same species [15], but its acrophase is 2.5-h advanced compared to *preproghrelin* expression. This lag might support that GOAT is likely acylating other substrates, apart from ghrelin, in the gastrointestinal tract. The existence of a rhythmic expression of all the components of the ghrelinergic system (ghrelin, GOAT and its main receptor) indicates that this system may be acting as an output of the circadian system in goldfish. Besides, the modulation of clock genes expression by ghrelin in goldfish tissues both *in vivo* [12] and *in vitro* [14] points to the ghrelinergic system as an input signal for the circadian system in fish.

The fact that the daily rhythms observed for *goat* mRNA levels in goldfish fed during the photophase are not displayed when food was delivered during the scotophase clearly indicates that the time of feeding is a critical external factor for the 24-h rhythmic expression of *goat* transcripts. Thus, in both central and peripheral tissues, such rhythmicity highly depends on whether feeding takes place during the light phase or the dark phase of the photocycle. Previous reports have described changes in plasma GOAT linked to feeding with a marked preprandial increase [35]. In terms of gene expression, periprandial changes in hypothalamus and gut are not so consistent in fish, but it seems that the lack of a meal increases *goat* expression in these locations [22,35], supporting the relevance of feeding in the daily profile of *goat* expression in two locations related to food intake regulation. In support of this relationship between feeding time and daily rhythm of *goat*, a previous report in mice has shown that *goat* exhibits a daily expression profile in stomach with a diurnal acrophase. Considering that these mice had free access to chow but showed a clear nocturnal food intake pattern, the peak of *goat* expression during the photophase in these mice is in accordance with our observations. In our study, fish also showed a food ingestion pattern according to food availability, but feeding time was not able to synchronize daily pattern of *goat* in goldfish brain and gut, although it is a potent synchronizer of key elements of the circadian system, such as some clock genes (own observations). Thus, it is possible that many other external cues may play key roles in the synchronization of daily profile of *goat* expression.

It is worthy to highlight that the rhythmic expression of *goat* is tissue-dependent, as such rhythms were observed in hypothalamus, intestinal bulb and pituitary, but not in telencephalon and vagal lobe. The existence of rhythmic profiles in hypothalamus and intestinal bulb, key regulators involved in food intake control, supports the important role of the ghrelinergic system as appetite regulator. This is in accordance with the anatomical observations on GOAT being expressed in the same brain areas as other feeding regulators, such as NPY and orexin [50], and on *ghs-r1a* being highly expressed in hypothalamic nuclei related to food intake regulation [15]. Concerning the pituitary, present results represent the first report for a rhythmic expression of *goat* in this gland in any vertebrate. The high expression of *goat* found in this master gland, together with the robust daily expression rhythms of *preproghrelin*, *goat* and *ghs-r1* in this tissue, emphasizes the relevance of the ghrelinergic system in the pituitary. The physiological importance of such observation is yet to be investigated, but it might be related with a circadian regulation of the actions of the ghrelinergic system in this gland (modulatory effect on the secretion of growth hormone), and with the role of ghrelin in reproductive functions [48, 49], also supported by the high expression of *goat* mRNAs in gonads. Together, present results suggest the existence of a circadian regulation of the acylating process of ghrelin in tissues importantly involved in physiological actions known to be regulated by ghrelin.

## Conclusions

The present study identifies the full-length sequence of goldfish ghrelin O-acyltransferase, revealing that this teleost has at least two highly homologous cDNAs, named goldfish *goat-V1* and *goat-V2*, encoding this enzyme. These two forms of *goat* would be generated by the transcription of three exons, the first of which differs from the exon 1 that is commonly transcribed in the rest of vertebrates and which we are referred to as exon 1’. Besides the fact that exon 1’ and not the typical exon 1 is to be transcribed in goldfish, *goat-V1* would be transcribed by regular splicing, while *goat-V2* would be generated by alternative 3’ splice site selection at exon 2. Results presented here offer novel data on the tissue-specificity and regulatory mechanisms underlying GOAT expression. Thus, we found that ghrelin exerts a negative feedback on both the expression of the gene encoding the enzyme and on the protein levels. Feeding time was also observed to be a key factor regulating *goat* mRNA levels, as a rhythmic expression was found in hypothalamus, pituitary and intestinal bulb when fish were fed at midday, but not when they were fed at midnight. The fact that GOAT might be synthesized in a rhythmically manner in the intestinal bulb, together with the observation that this enzyme colocalizes its main substrate ghrelin in enteroendocrine cells, might indicate an important local regulation of ghrelin activation. Collectively, data presented in this work offer novel data on the characterization of GOAT in a non-mammalian vertebrate and represent an important step in the understanding of the activation of ghrelinergic system in fish.

## Supporting information

S1 Fig Exon-intron structure of the *goat* gene in representative teleosts from the Cyprinidae family: the zebrafish (*Danio rerio*), the horned golden-line barbel (*Sinocyclocheilus rhinocerous*) and the common carp (*Cyprinus carpio*), and the proposed structure for goldfish (*Carassius auratus*). Exons are indicated by boxes and introns by lines. The length (pb) of exons and introns is indicated inside the boxes (exons) or above lines (introns).
(JPG)

S2 Fig Alignment of the beginning of the nucleotide sequences of *goat* from various cyprinids to show the high degree of conservation of the exon 1’. Multiple sequence alignment was conducted using Clustal W2 (http://www.ebi.ac.uk/Tools/msa/clustalw2/) and edited using the BioEdit Sequence Alignment Editor. The main part of intron 1’, exon 1 and intron 1 was omitted for a better comprehension of figure; this is indicated by suspension points (…). Dashed lines represent voids introduced to optimize the alignment. Identical nucleotides among sequences are colored. GT/AG indicates the putative donor/acceptor sites for splicing, and ①② the start codons (ATG) for goldfish transcripts. The common name of the species used for the alignment is given on the right side, and the species names and GenBank accession numbers of genomic sequences are as follows: common carp, *Cyprinus carpio*, (1) LHQP01003245.1(80257–80106….78407–78390) and (2) LHQP01015814.1(57963–58120….64869–64886); horned golden-line barbel, *Sinocyclocheilus rhinocerous*, (1) NW_015642610.1(2326737–2326895…2328839–2328856) and (2) NW_015656585.1(648128–647968….641970–641953); zebrafish, *Danio rerio*, CABZ01049031.1(6992–7150…18211–18228). Sequences for goldfish, *Carassius auratus*, are from cDNA: (V1) KX953158, (V2) KX953159 and (V3) GBZM01002161.1(1008–800).
(TIF)

S3 Fig Alignment of the nucleotide sequences of the exon 1 of *goat* from various cyprinids. Multiple sequence alignment was conducted using Clustal W2 (http://www.ebi.ac.uk/Tools/msa/clustalw2/) and edited using the BioEdit Sequence Alignment Editor. Dashed lines represent voids introduced to optimize the alignment. Identical nucleotides among sequences are colored. The common name of the species used for the alignment is given on the right side, and the species names and GenBank accession numbers of genomic sequences are as follows: common carp, *Cyprinus carpio*, (1) LHQP01003245.1(78599–78478) and (2) LHQP01015814.1(64651–64772); horned golden-line barbel, *Sinocyclocheilus rhinocerous*, (1) NW_015642610.1(2328647–2328768) and (2) NW_015656585.1(642129–642068); zebrafish, *Danio rerio*, CABZ01049031.1(18010–18134).
(TIF)

S4 Fig Alignment of the amino acid sequences of GOAT from various vertebrates used to create the phylogenetic tree shown in Fig 4. Multiple sequence alignment was conducted using Clustal W2 (http://www.ebi.ac.uk/Tools/msa/clustalw2/) and edited using the BioEdit Sequence Alignment Editor. Dashed lines represent voids introduced to optimize the alignment. Identical amino acids among sequences are colored. In common carp-2 GOAT: Δ_n_, indicates frameshift detetion; n, number of bases deleted; X, nonsense mutation. When nucleotide sequences are used, they were translated into amino acids using Wise2 (http://www.ebi.ac.uk/Tools/psa/genewise/). The common name of the species used for the alignment is given on the right side, and the species names and GenBank accession numbers are as follows: alligator, *Alligator sinensis*, XP_006035341.1; Asian arowana, *Scleropages formosus*, JARO02002481.1(36315–36436….36746–36978….37427–38368); Atlantic herring, *Clupea harengus*, JZKK01021833.1(58124–58006….52933–52709….50211–49272); Atlantic salmon, *Salmo salar*, XP_014016526.1; channel catfish, *Ictalurus punctatus*, XP_017306886.1; chimpanzee, *Pan troglodytes*, ENSPTRT00000037288; cock, *Gallus gallus*, NP_001186218.1; coelacanth, *Latimeria chalumnae*, BK009986; common carp, *Cyprinus carpio*, (1) LHQP01003245.1(78599–78478….78401–78176…78096–77163) and (2) LHQP01015814.1(64651–64772….64875–65084….65162–65417….67215–67900); damselfish, *Stegastes partitus*, XP_008292386.1; elephant shark, *Callorhinchus milii*, BK009985; frog, *Xenopus tropicalis*, XP_002936505.2; goldfish, *Carassius auratus*, (V1) APD26025 and (V2) APD26026; horned golden-line barbel, *Sinocyclocheilus rhinocerous*, (1) XP_016428796.1 and (2) XP_016383356.1; human, *Homo sapiens*, ACB05873.2; Japanese eel, *Anguilla japonica*, AVPY01018663.1(5671–5450….5379–5155….4515–3518); lizard, *Anolis carolinensis*, XP_003224702.1; Mexican cavefish, *Astyanax mexicanus*, XP_007253942.1; mouse, *Mus musculus*, ACB05874.1; rabbit, *Oryctolagus cuniculus*, ENSOCUT00000014851; rainbow trout, *Oncorhynchus mykiss*, CDQ71181.1; rat, *Rattus norvegicus*, ACB05875.1; red pirahna, *Pygocentrus nattereri*, MAUM01004312.1 (861–985…1063–1287…2692–3625); spotted gar, *Lepisosteus oculatus*, BK009987; stickleback, *Gasterosteus aculeatus*, AANH01001771.1 (95917–95796…95445–95215…95100–94149); striped bass, *Morone saxatilis*, JTCL01001059.1(43688–43809….44568–44792….45768–46719); tilapia, *Oreochromis niloticus*, AERX01036891.2(7728–7607….6577–6353….4935–3987); wild boar, *Sus scrofa*, ADI55170.1; yellow croacker, *Larimichthys crocea*, XP_010729215.1; yellowbelly rockcod, *Notothenia coriiceps*, AZAD01045919.1(5788–5667….5115–4891….4240–3295); zebra mbuna, *Maylandia zebra*, XP_014262684.1; zebrafish, *Danio rerio*, ACB05876.1.
(PDF)

S5 Fig Representative original uncropped Western blot corresponding to the bands shown in Fig 8. Blot shows GOAT levels in goldfish intestinal fragments exposed (or not, control) to different concentrations of goldfish acylated ghrelin during 30 min. Molecular size marker is shown in the left lane, and weights of each protein band (in kDa) is indicated. The goldfish GOAT has a molecular weight of around 45 and 42 kDa (GOAT-V1 and GOAT-V2, respectively).
(TIF)
