# Supplementary figures and images for: Characterization of Ghrelin O-Acyltransferase (GOAT) in goldfish (Carassius auratus)
Source: PLoS One. 2017 Feb 8;12(2):e0171874. doi: 10.1371/journal.pone.0171874 (PMC5298278; doi:10.1371/journal.pone.0171874)

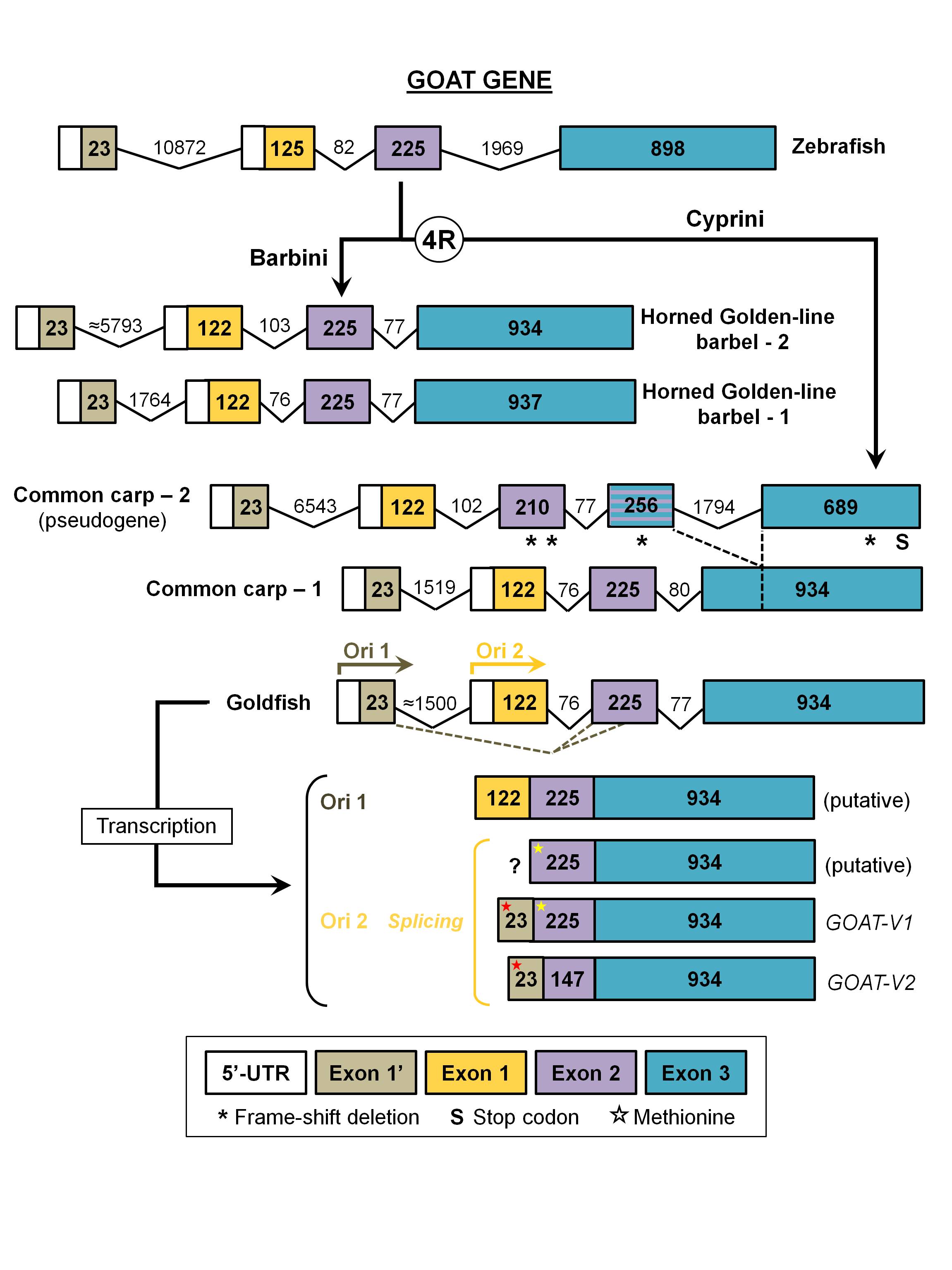

Supplement: S1 Fig — Exons are indicated by boxes and introns by lines. The length (pb) of exons and introns is indicated inside the boxes (exons) or above lines (introns). (JPG) [file pone.0171874.s001.jpg]

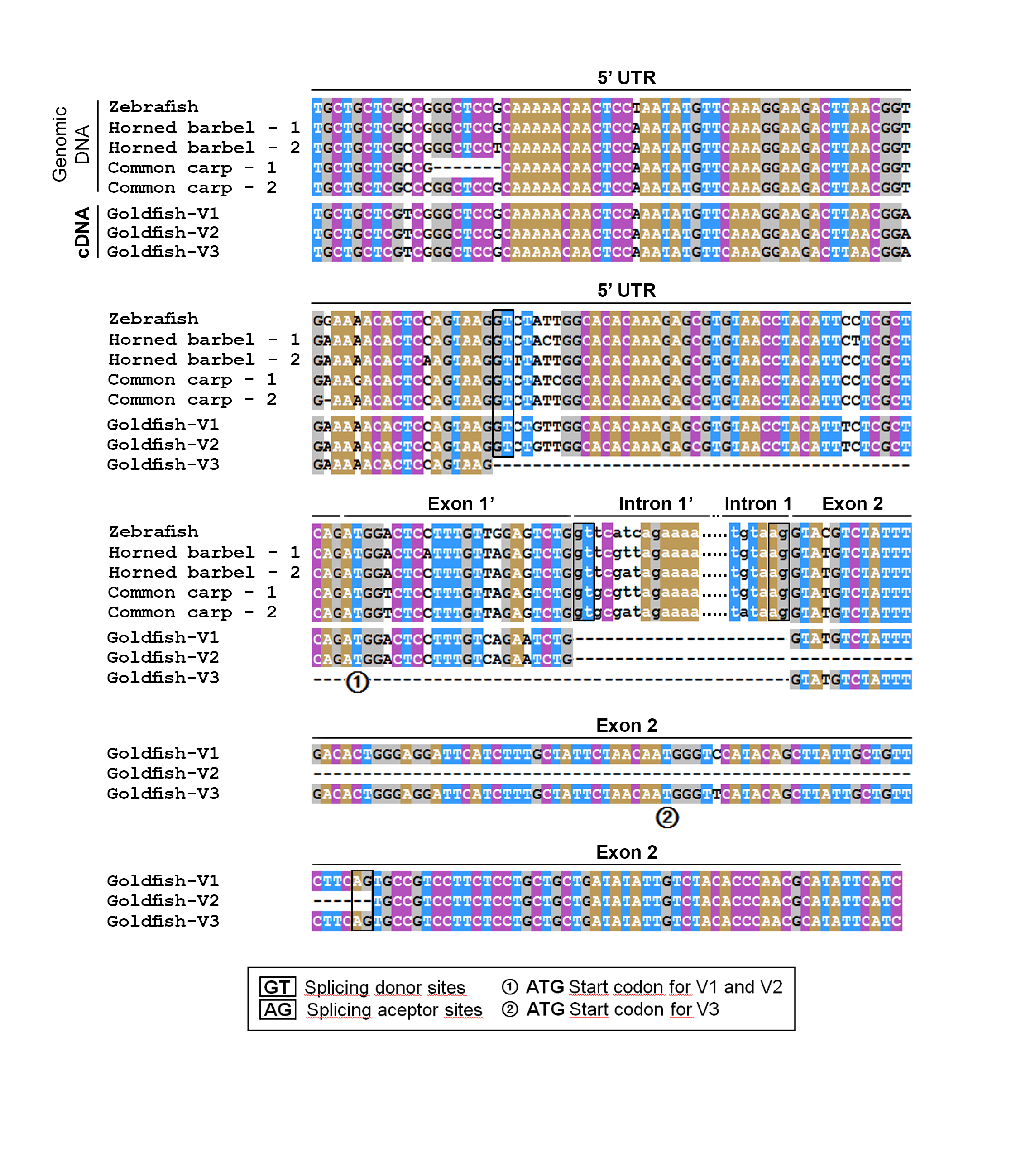

Supplement: S2 Fig — Multiple sequence alignment was conducted using Clustal W2 (http://www.ebi.ac.uk/Tools/msa/clustalw2/) and edited using the BioEdit Sequence Alignment Editor. The main part of intron 1’, exon 1 and intron 1 was omitted for a better comprehension of figure; this is indicated by suspension points (…). Dashed lines represent voids introduced to optimize the alignment. Identical nucleotides among sequences are colored. GT/AG indicates the putative donor/acceptor sites for splicing, and ①② the start codons (ATG) for goldfish transcripts. The common name of the species used for the alignment is given on the right side, and the species names and GenBank accession numbers of genomic sequences are as follows: common carp, Cyprinus carpio, (1) LHQP01003245.1(80257–80106….78407–78390) and (2) LHQP01015814.1(57963–58120….64869–64886); horned golden-line barbel, Sinocyclocheilus rhinocerous, (1) NW_015642610.1(2326737–2326895…2328839–2328856) and (2) NW_015656585.1(648128–647968….641970–641953); zebrafish, Danio rerio, CABZ01049031.1(6992–7150…18211–18228). Sequences for goldfish, Carassius auratus, are from cDNA: (V1) KX953158, (V2) KX953159 and (V3) GBZM01002161.1(1008–800). (TIF) [file pone.0171874.s002.tif]

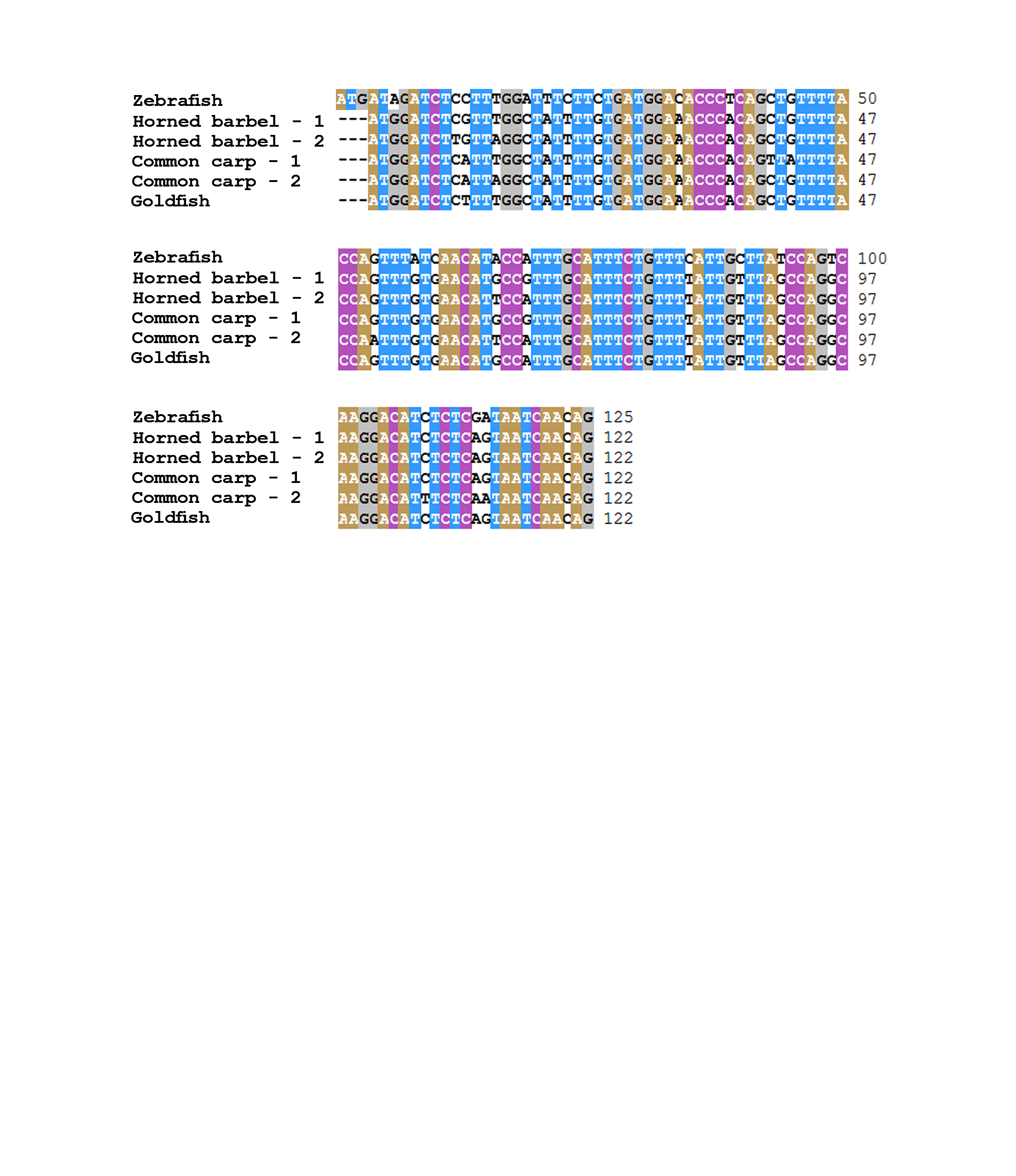

Supplement: S3 Fig — Multiple sequence alignment was conducted using Clustal W2 (http://www.ebi.ac.uk/Tools/msa/clustalw2/) and edited using the BioEdit Sequence Alignment Editor. Dashed lines represent voids introduced to optimize the alignment. Identical nucleotides among sequences are colored. The common name of the species used for the alignment is given on the right side, and the species names and GenBank accession numbers of genomic sequences are as follows: common carp, Cyprinus carpio, (1) LHQP01003245.1(78599–78478) and (2) LHQP01015814.1(64651–64772); horned golden-line barbel, Sinocyclocheilus rhinocerous, (1) NW_015642610.1(2328647–2328768) and (2) NW_015656585.1(642129–642068); zebrafish, Danio rerio, CABZ01049031.1(18010–18134). (TIF) [file pone.0171874.s003.tif]

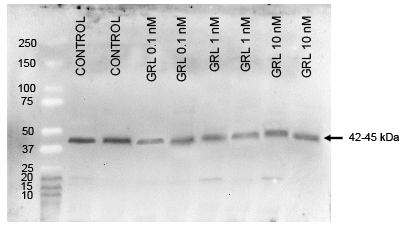

Supplement: S5 Fig — Blot shows GOAT levels in goldfish intestinal fragments exposed (or not, control) to different concentrations of goldfish acylated ghrelin during 30 min. Molecular size marker is shown in the left lane, and weights of each protein band (in kDa) is indicated. The goldfish GOAT has a molecular weight of around 45 and 42 kDa (GOAT-V1 and GOAT-V2, respectively). (TIF) [file pone.0171874.s005.tif]
