# Supplementary material for: Characterization of Ghrelin O-Acyltransferase (GOAT) in goldfish (Carassius auratus)
Source: PLoS One. 2017 Feb 8;12(2):e0171874. doi: 10.1371/journal.pone.0171874 (PMC5298278; doi:10.1371/journal.pone.0171874)
Supplement: S4 Fig — Multiple sequence alignment was conducted using Clustal W2 (http://www.ebi.ac.uk/Tools/msa/clustalw2/) and edited using the BioEdit Sequence Alignment Editor. Dashed lines represent voids introduced to optimize the alignment. Identical amino acids among sequences are colored. In common carp-2 GOAT: Δn, indicates frameshift detetion; n, number of bases deleted; X, nonsense mutation. When nucleotide sequences are used, they were translated into amino acids using Wise2 (http://www.ebi.ac.uk/Tools/psa/genewise/). The common name of the species used for the alignment is given on the right side, and the species names and GenBank accession numbers are as follows: alligator, Alligator sinensis, XP_006035341.1; Asian arowana, Scleropages formosus, JARO02002481.1(36315–36436….36746–36978….37427–38368); Atlantic herring, Clupea harengus, JZKK01021833.1(58124–58006….52933–52709….50211–49272); Atlantic salmon, Salmo salar, XP_014016526.1; channel catfish, Ictalurus punctatus, XP_017306886.1; chimpanzee, Pan troglodytes, ENSPTRT00000037288; cock, Gallus gallus, NP_001186218.1; coelacanth, Latimeria chalumnae, BK009986; common carp, Cyprinus carpio, (1) LHQP01003245.1(78599–78478….78401–78176…78096–77163) and (2) LHQP01015814.1(64651–64772….64875–65084….65162–65417….67215–67900); damselfish, Stegastes partitus, XP_008292386.1; elephant shark, Callorhinchus milii, BK009985; frog, Xenopus tropicalis, XP_002936505.2; goldfish, Carassius auratus, (V1) APD26025 and (V2) APD26026; horned golden-line barbel, Sinocyclocheilus rhinocerous, (1) XP_016428796.1 and (2) XP_016383356.1; human, Homo sapiens, ACB05873.2; Japanese eel, Anguilla japonica, AVPY01018663.1(5671–5450….5379–5155….4515–3518); lizard, Anolis carolinensis, XP_003224702.1; Mexican cavefish, Astyanax mexicanus, XP_007253942.1; mouse, Mus musculus, ACB05874.1; rabbit, Oryctolagus cuniculus, ENSOCUT00000014851; rainbow trout, Oncorhynchus mykiss, CDQ71181.1; rat, Rattus norvegicus, ACB05875.1; red pirahna, Pygocentrus natterer [file pone.0171874.s004.pdf]

```
                                       Exon 1                                 Exon 2
Channel catfish       --MDLLWMIFNQNPQLAYQLFTIPLAFLFYSLATQGYLTLFNRHICLALGGFIVAILTMGPYSTLLFITFITFVLLVRFM 78
Red piranha           --MDLLWIIFNQNPQLAYQLFTIPFAWLFYSLATRGYLTLINRYMFLTLGGFILAVLTMGPYSLLLFITATKFVLLVHFV 78
Mexican cavefish      --MDFLWNIFNQNPQLAYQFYLIPLALVFYGLTMTGYLTLINRYICLMFGGAILAVLTMGPYSILLFITATMFVFLVHFV 78
Zebrafish             -MIDLLWISSDGHPQLFYQFINIPFAFLFHCLSSQGHLSIINRYVYLAMGGFMLAIATMGPYSSLLFLSAIKLLLLIHYI 79
Common carp - 2       --MDLIRLFCDGNPQLFYQFVNIPFAFLFYCLARQGHFSIIKRYVYLTLGGFILVIVTVGPYSLLLFFSAVLLLLLIRCL 78
Horned barbel - 2     --MDLVRLFCDGNPQLFYQFVNIPFAFLFYCLARQGHLSVIKRYVYLTLGGFILAIVTMGPYSLLLFFSAVLLLLLIRCL 78
Goldfish-V1           --MDSFVRIW--------------------------------YVYLTLGGFIFAILTMGPYSLLLFFSAVLLLLLIYCL 45
Goldfish-V2           --MDSFVRIC--------------------------------------------------------AVLLLLIYCL 19
Horned barbel - 1     --MDLVWLFCDGNPQLFYQFVNMPFAFLFYCLARQGHLSVINRYVYLTLGGFIFAIVTVGPYSLLLFFSAVLLLLLIYCL 78
Common carp - 1       --MDLIWLFCDGNPQLFYQFVNMPFAFLFYCLARQGHLSVINRYVYLTLGGFIFAIVTMGPYSLLLFFSAVLLLLLIYCL 78
Spotted gar           --MDLSWCVFDLHPQIVYQFSALPFALLFYFFTKLKCLSVISRYIYLLLGGCVLATVTMGQYCLIVLTPAATFVLLLLSV 78
Japanese eel          --MDLKLMVFQQNSQLIYQFLPFPFALLYYIILARQGCLSSTNRYVYLALGGCVLAMVSMGPYCWLVLVPVGASAVLAPSA 78
Asian arowana         --MDFIRWLFRQSPHLLYQFFSIPFALLFYILARLDCLTLTHRYVYLMSGGCVLAAVTMGPYACLLLVPAFWAVLLFCSL 78
Atlantic herring      ---MASIWDFSGSAQLMYQILSLPFALIFYCLATNGYLTLTQRYLYLGVGGFSLAVISMGWYSIVLFVSVIMSALLIYYL 77
Atlantic salmon       --MEPIQWLCEQHPFLMYQCFSIPFAFLFYVLAKQGCLSLTYRYLFLASGGCILAVVTMGVYSLLLLVSTVIFVLLVCSL 78
Rainbow trout         --MEPIQWLCEQHPFLTYQCFSIPFAFLFYVLAKQGCLSLTYRYLFLASGGCILAIVTMGVYSLLLFISTVIFVLLVCSL 78
Stickleback           --MGLMSSFWERHQFLMHQCFSLPFAFLFFFLAKRGYLSLTYRYLFVSAGGCVLAVVTMGVYSSLLFASALVFVLLVVSV 78
Zebra mbuna           --MGSIGWSWEQHRFLMYQCLSLPFAFLFYFLAKRGHLSLTYRYLFVSAGGSVLAVFTMGIYSTLLFTSTLAFVLLVCCV 78
Tilapia               --MGSIGWSWEQHRFLMYQCLSLPFAFLFYFLAKRGHLSLTCRYLFVSAGGSVLAVITMGIYSTLLFTSTLAFVLLVCCV 78
Damselfish            --MGSIGWLWEQHQLLMHQCCSLPFAFLFYFLSKRRYLSLTYRYLFVSVGGCVLAVMTMGIYSLILFTSTFVFVLLVLSV 78
Yellowbelly rockcod   --MGLMSSLWEQHQFLMHQCFSLSFAFLFYILAKRGYLGLTYRYLFVSVGGCVLAVVTMGVYSLLLFTSTSVFILLVCSV 78
Yellow croaker        --MGLMNWLWEHHQFLMHQCFSLPFAFLFYFLAKWGYLSLRCRYLFVSIGGCVLAVVTMGIYSTLLFTSTFAFILLVCSV 78
Striped bass          --MGSMNWLWEHHQFLMHQYFLLPFAFLFYFLAKLGFLSLTYRYLFVAIGGCVLAVVTMGIYSALLFTSTFVFILLVCSV 78
Elephant shark        MDLEFLQFQLDFYPVTMYQLMSVPLAFMFYQLSIKGHLPLTARYLFLVFGGLLVMCVSMGPYAALVLIPALSSVMMLHSL 80
Frog                  --MDY--LQLVFHPVALYQLAAFPWAFLYGYLSSFGHLPITPRFIYLLFGGIVLSCVSMGPYAVVIFIPALGSIVLFHII 76
Coelacanth            --MDLSIVLSYVHPVTIYQLVIFPFAVVFYYLCIHGSLSVTARYAFLLVGGFLLSNIAMGTFSLLVLIPAVLSVIMMHSI 78
Cock                  --MR-WADLLILLPAAWYQLAAFLFAALFHYLCALGHLSLTSRYIFLLAGGCLLAGTAMGSYATLLLIPAVSSVLMFLLV 77
Lizard                --MN-WESPFLFQATAFYQLAALPFAFLFHYLCCCTNLSINSRYIFLLVGGFILACAAIGCYALLVFVSAFCSLAIIHSA 77
Alligator             --ME-WADPFLLNPTILYQMLAFPFAVLFHYLCSFGYLSPSARYVVLLVGGFLIACAAMGLYALLLFIPALCSVAIFHSV 77
Wild boar             --MD-WLQLFFLHPVLFYQGAAFPFALLFNYLCITDSFSTRARYLFLLAGGGALAVAAMGAFAVLVFVPALGAVVLIHSL 77
Mouse                 --MD-WLQLFFLHPLSFYQGAAFPFALLFNYLCILDTFSTRARYLFLLAGGGVLAFAAMGPYSLLIFIPALCAVALVSFL 77
Rat                   --MD-WLQFFFLHPVSLYQGAAFPFALLFNYLCITESFPTRARYLFLLAGGGALALAAMGPYALLIFIPALCAVAMISSL 77
Chimpanzee            --ME-WLRLFFLHPVSFYQGAAFPFALLFNYLCIMDSFSTRARYLFLLAGGGALAVAAMGSYAVLVFTPAVCAVALLCSL 77
Human                 --ME-WLWLFFLHPISFYQGAAFPFALLFNYLCIMDSFSTRARYLFLLAGGGALAVAAMGSYAVLVFTPAVCAVALLCSL 77
Rabbit                --MD-WLQLFFLHPVSLYQGAAFPVALLFNYLCIWDSFSARARYLFLLAGGGTLAVAAMGLYAMLVFTPAVCAVFLVSSL 77

                              Exon 2                          Exon 3
Channel catfish       EPVHIHYWIFGLQMCWQTLWHFYMQYQHYWLQEPPDSRFVLAMSALMLLSQRVTSVSMDLQEGKVIRNFRGSFQ------ 152
Red pirahna           EPLHVHQWILGLQMCWQTLWHFYTQYQQYWLQKPADSRLLLAMSALMLLSQRVTSVSMDIQEGKVAKAFRGSYR------ 152
Mexican cavefish      EPVDVHHWIFGLQMCWQTFWHFYMQYQQYWLQETADSRLLLATSALMLLSQRVTSVSMDLQERKVCRPFGRTYQ------ 152
Zebrafish             HPMHLHRWIILGLQMCWQTCWHLYVQYQIYWLQEAPDSRLLLAISALMLMTQRISSLSLDFQEGTISNQ----------- 147
Common carp - 2       QQMHLLDVM Δ5LQMCWETCWHLYIΔ10--YWLQETPDFRLLLATSALMLMTQRVSSLALΔ8- QGTVTSPFQNSSR------ 146
Horned barbel - 2     HPMHIHYWTLGLQMCWQTCWHLYIQYQLYWLQETPDFRLLLATSAIMLMTQRVSSLSLDLQEGTVTSPFQNSSR------ 152
Goldfish-V1           HPTHIHHWTLGLQMCWQTVWHLYIQYQLYWLQETPDSRLLLAISALMLMTQRVSSLSLDLQEGTITSSFQNSSQ------ 119
Goldfish-V2           HPTHIHHWTLGLQMCWQTVWHLYIQYQLYWLQETPDSRLLLAISALMLMTQRVSSLSLDLQEGTITSSFQNSSQ------ 93
Horned barbel - 1     QPTHIHQWMLGLQMCWQTFWHLYIQYQLYWLQETPDSRLLLAISALMLMTQRVSSLSLDLQEGTVTSPFQNSSQ------ 152
Common carp - 1       HPTRIHQWTMGLQMCWQTFWHLYIQYQLYWLQETPDSRLLLAISALMLMTQRVSSLSLDLQEGTVTSPFQNSSQ------ 152
Spotted gar           HPQHVHFWGFFVQMCWQTLWHLLIQYKEHWLQESIDNRLLVAISSLMLLTQRVTSLSMDIQEGKLQSLQTKAQSS----- 153
Japanese eel          RSLHAHTCVFGAQMCWQTGWHLFIQYKEYWLQEPVDTRLLIAVSSLMLLTQRITSVSMDLQEGKLTGTQNVGSATSALAP 158
Asian arowana         RPWNVHPWVLGTLMCWQTFWHFLIQYQEYWLLQPSNTRLLLTMSAMMLLTQRATSVSMDLQEGKAVPPTCV--------- 149
Atlantic herring      DSQIIHPWIFTVQMFWQSLWHVFLLIRLLWHEEPTDHRLLLMVSSLMLLTQRVTSVSMDLEEGKVILPPRFKGR------ 151
Atlantic salmon       SPERVHPWVFGLQMGWQTFWHLLIQYREYYLNEPTDSRLLLSVSSLMLLTQRVTSVSMDLQDGGRVTLM----------- 147
Rainbow trout         SPERVHPWVFGLQMGWQTFWHLLIQYREYYLNEPTDSRLLLSMSSLMLLTQRVTSVSMDLQDGGRVTLM----------- 147
Stickleback           DAGRVHAWVFGIQMLWQTFWHLLIQYREYYLQEPVSLRLFLAVSSLMLLTQRITSLSMDLQERRVRPPAAAAPQQ----- 153
Zebra mbuna           DHSCIHTWAFVMQMLWQTFWHLLIQYREYYLHERVSIRLFLAVSTLMLLTQRITSVSLDLQEKRVVLTP---SKR----- 150
Tilapia               DHSCIHTWAFLMQMLWQTFWHLLIQYREYYLHEPVSIRLFLAVSTLMLLTQRITSVSLDLQEKRGVLTP---SKR----- 150
Damselfish            DPGCVHVWAFGVQMLWQTFWHLFIQYKEYYLHEPVSIRLFLAASSLMLLTQRITSVSMDLQETQVLLTCNAITRR----- 153
Yellowbelly rockcod   DLRHVHTWVFTVQMLWQTFWHLLIQYREYYLHEPVSIRLFVAVSSLMLLTQRITSLSMDLQEKRVVLTFKASSKR----- 153
Yellow croaker        DSSSIHAWVFSMQMLWQTFWHLFIQYKEYYLHEPVCIRLFLAVSSLMLLTQRITSLSMDLQEKRVLTTFNASSKR----- 153
Striped bass          DPSSVHIWAFSIQMLWQTFWHLLIQYREYYLHEPVCIRLFLAMSSLMLLTQRITSVSMDLQEKRVMLTFNASSKR----- 153
Elephant shark        RAMTVHKLIFFTQMSWLSLCHLWLHYKDYYLQEPTDTKFLIALSSLMLLIQRVTSVSLDVHEGKVTIAKMQRH------- 153
Frog                  SWQSVHWWALALQMVWQTACHLWLLYKEYYLQEEITLRLSIMISALMLLTQKITTLALDIHERKVRIIPVDGGMK----- 151
Coelacanth            SPQSVHRWVFLIQMLWQTLCHLWLQYKEHSMQEITRIRFIITISSLMLLTQRVTTLALDIHEGKIKIATRSSYL------ 152
Cock                  SPAYVHTWVFSLQMCWQTLCHLGLG---SLVLESQDTRPAVTLSAIMLLTQKVTSLALDIHEGTVLPQPGQ--------- 145
Lizard                DLQQVHQWAFCFQMLWQTLCHFWLQYKEYYLQETTCPRFSITLSVLMLMTQRITSLTLDIHEGKVRLWLPHA-E------ 150
Alligator             SPLRVHTWVFVFQMSWQTLCHLGLHYREHYLQEAPCIRLSIALSSLMLLTQKVTSLALDIHEGTVTVTFEHRRR------ 151
Wild boar             GPRHVHRPTFLFQMSWQTLCHLGLHYTEYYLQESPSTRFCITLSSFMLLTQRVTSLSLDIFEGKVEVASEAVGS------ 151
Mouse                 SPQEVHRLTFFFQMGWQTLCHLGLHYTEYYLGEPPPVRFYITLSSLMLLTQRVTSLSLDICEGKVEAPRRGIRS------ 151
Rat                   SPQEVHGLTFFFQMGWQTLCHLGLHYKEYYLCEPPPVRFYITLSSLMLLTQRVTSLSLDISEGKVEAAWRGTRS------ 151
Chimpanzee            APQQVHRWTFCFQMSWQTLCHLGLHYTEYYLQEPPSVRFCITLSSLMLLTQRVTSLSLDICEGKVEAASGGFRS------ 151
Human                 APQQVHRWTFCFQMSWQTLCHLGLHYTEYYLHEPPSVRFCITLSSLMLLTQRVTSLSLDICEGKVKAASGGFRS------ 151
Rabbit                SPPEVHRWTFLFQMGWQTLCHLGLQYTEHYLQEPPSMRFCIALSSLMLLTQRVTSLSLDICEGKVEAASGGVGS------ 151
```

Exon 3

```
Channel catfish        --------SQVVCLAPFMSYTLYFPALLIGGPLCPFNTYVNFVEQISVNRPPSPL-TIL-PW-------KMLQVLLLLVLK 215
Red pirahna            --------SQAISLIPFLSYSLYFPALLGGPLCSFTTYVTFVEQISIRPPPSPL-AIL-PW-------KILRVLVVLVLK 215
Mexican cavefish       --------SKICSLIPFLSYTLYFPALLGGPLCPFKSYVAFVEQMGCRTPPSPY-AIV-LW-------KILQVLFLLVLK 215
Zebrafish              -----------SILIPFLTYSLYFPALLGGPLCSFNAFVQSVERQHTSMT--SY-LGNLTSK-------ISQVIVLVWIK 206
Common carp - 2        ---------GLSVLIPFLSYSLDFPALLEGPLCSFNTFVESVRQLSMTSPLASY-LGRLSKIIFSYSFHLWQVVVLVWIK 216
Horned barbel - 2      ---------GLSVLIPFLSYSLNFPALLGGPLCSFNTFVESVRQISVTSPLASY-LGRLTSK-------MLQVVVLVWIK 215
Goldfish-V1            ---------DLSVLIPFLSYSLNFPALLGGPLCSFNTFVQSVRQMSVTPPLTPY-LRRLTSK-------ILQVIILVWIK 182
Goldfish-V2            ---------DLSVLIPFLSYSLNFPALLGGPLCSFNTFVQSVRQMSVTPPLTPY-LRRLTSK-------ILQVIILVWIK 156
Horned barbel - 1      ---------DLSILIPFLSYSLNFPALLGGPLCSFNTFVQSVRQMSVTPPLASY-LRRLTSK-------LLQVLVLVWIK 215
Common carp - 1        ---------DLSVLIPFLSYSLNFPALLGGPLCSFNTFVWSVRQMSVTPPLGSY-LRRLTSK-------ILQVLVLVWIK 215
Spotted gar            ------VRYNFPLWIPYLSYTLFFPALLGGPLCSFSYFRSFVEQNGLSSIPF---PLGAV------LQKTLLVLSLEGAK 218
Japanese eel           HLAGTHSVGSATSALQYLSYTLYFPGLLGGPLCSFQQFVSFVEQRVSP---APQ-PLYAV------CCKVCWALGLQWGK 228
Asian arowana          -----------AGALPYLSYTLYFPGLLGGPLCSFSQFVRFVEKSGSVPPPPPS-PLRPV------LLKCLMAFSLEWAR 211
Atlantic herring       --------VYSSHLLPFACYSLSFVFLLGGPLCPYDQFVSFVQHIQQNPPPS---PLNRV------SLRLLWVLILEGIK 214
Atlantic salmon        --------IQCRVFLPLISYALNFTALLGGPLSSFDQFVYFVEQITISPPPQ---PLSVI------SYKGFQVLSLEWAR 210
Rainbow trout          --------TQCRVFLPLISYALNFTALLGGPLSSFDQFVYFVEQTTISPPPQ---PLSVI------SYKGFQVLSLEWAR 210
Stickleback            --------EARAALLPLVSYILNFTTLLGGPLCPYGRFVSLMTGISADPPPE---PLRAV------FLKLTQVLPMELVR 216
Zebra mbuna            --------QTCVTLLPLISYILNFTTLLGGPLGSYGQFITLMEGINLTSPPS---PPGVV------FLKLMQVLMLEWVR 213
Tilapia                --------QACVTLLPLISYILNFTTLLGGPLGSYGQFITLMEGINLTSPPR---PLGVV------FLKLMQVLMLEWVR 213
Damselfish             --------QSRVMILPLISYSLSFTTLLGGPLCSYSRFVLLMEGLGLSAPPS---PLAVV------LLKLVQVLLLEGVR 216
Yellowbelly rockcod    --------KACVMLLPLISYILNFTTLLGGPLCSYRHFVSLMAGISLHAPPN---PLGVV------LLKLMQIIMLECVK 216
Yellow croaker         --------NRRVMLLPLISYILNFTTMLGGPLCSYSRFVTLMAEIRFNHPPN---PLGVV------LIKLTQVMLLECLR 216
Striped bass           --------KACVMLLPLISYTLNFTTLLGGPLCSYSRFVSLMAGIRLNPPPN---PQGVV------FLKLIQVLLLECLR 216
Elephant shark         ----HSSSEFLRCLLPHISYVLYFPALLGGPLCSFQMFQIHMENLKKSQQKRNANSIWPF------LKKCLLVFIMDRLR 223
Frog                   ---NWFFSGSAHNILIFLSYLLFFPALLGGPLCSFVEFHHHVTVAPRCNYL----CFKQV------AKGFFFALILQMLR 218
Coelacanth             ---NNSILQYLHNLLPYFSYMIYFPALLGGPLLSFQLFKIHIETSGVKCTEN--CSLP-V------TKKFCFFLALELLK 220
Cock                   --------GLLQRALPLCSYLLFFPALLGGPLCSFSKFQAQVMSLGAVPC-----PLRAV------CWRYLGVLVLQVLR 206
Lizard                 ---KGPLERHLHQALPVCTYLLSFPTLLGGPLCSFNRFQTWIRCSRRTSFSQ--CSLWAA------IQKALGALMLGFLK 219
Alligator              ---K----EPLLGALPFCSYLLFFPALLGGPLCSFKRFQAQILCSSLPYFT---QSLWVA------GQKSLWALTLQLLR 215
Wild boar              ---ESSLPKRLWKALPYCSYLLFFPALLGGPLCSFRRFQSHVQGPRSWDPR---HSLWAL------TCQGLQIVGLECLK 219
Mouse                  ---KSSFSEHLWDALPHFSYLLFFPALLGGSLCSFRRFQACVQRSSSLYPS---ISFRAL------TWRGLQILGLECLK 219
Rat                    ---RSSLCEHLWDALPYISYLLFFPALLGGSLCSFQRFQACVQRPRSLYPS---ISFWAL------TWRGLQILGLECLK 219
Chimpanzee             ---RSSLSEHVCKALPYFSYLLFFPALLGGSLCSFQRFQARVQGSSALHPR---HSFWAL------SWRCLQILGLECLN 219
Human                  ---RSSLSEHVCKALPYFSYLLFFPALLGGSLCSFQRFQARVQGSSALHPR---HSFWAL------SWRGLQILGLECLN 219
Rabbit                 ---RRSLAEHLGKALPYLSYLLFFPALLGGPLCSFQRFQARVQRSSSLGPK---HCFWVL------SWRGLQILGLESLK 219
```

Exon 3

```
Channel catfish        FLLTGVLQSSIF---SLS----SSPSILWIWIFSLVLRLTYYVHWKISECVNNAAGLGFSGYSTTG-GALWNGLSDGDAF 287
Red pirahna            YLFSSFLQLSIF---RLSS-PHDSPGILWIWILSLVLRLNYYAHWKISECLNNAAGLGFSGCGPNG-GTLWDGLSDGDPW 290
Mexican cavefish       YLLSSFLQLSIA---NLSS-SQESPGLLWVWIFSLVLRLNYYTHWKISECVNNAAGLGYCKGCLNG-GELWNGLSDGNPL 290
Zebrafish              QLFSELLKSATF---NIDS---VCLDVLWIWIFSLTLRLNYYAHWKMSECVNNAAGLGVYFHKHSG-QTSWDELSDGSVL 279
Common carp - 2        YPLKELLKSVTF---TVNS-PCICQNIIWIWVLLLLLKMNCYAHWKVSECVNSAAGMGFHGYSHSG-KSSWDEFSDGSPW 291
Horned barbel - 2      YPLKELLKSITF---TVNS-PCVCQNIIWIWVLSLLLKMNYYAHWKLSECVNNAAGMGFHAYSRSG-KSSWDGFSDGSPW 290
Goldfish-V1            YPLKELLKSITF---RVNS-PCVCQNIIWIWILSLLLKMNYYAHWKVSECVNNAAGIGFHGYSHSG-KKSWDGFSDGSPW 257
Goldfish-V2            YPLKELLKSITF---RVNS-PCVCQNIIWIWILSLLLKMNYYAHWKVSECVNNAAGIGFHGYSHSG-KKSWDGFSDGSPW 231
Horned barbel - 1      YPLKELLKSITF---RVNS-PCVCQNIIWIWILSLLLKMNYYAHWKVSECVNNAAGIGFHGYSHSG-KKSWDGFSDGSPW 290
Common carp - 1        YPLKELLKSFTF---RVNS-PCVCQNKIWIWILSLLLKMNYYAHWKVSECVNNAAGIGFHGFSHSG-KKSWDGLSDGSPW 290
Spotted gar            TFLRCHLSSNSL---SQAH-FNVLEGILFIWGISLLYKLSYYSHWTLSESLNNAAGLGFRGYNKHG-NPLWDALSDGNIL 293
Japanese eel           CVLVDVMERYSP---GLAQ-LSAVQSTLWVLGLALVFRLSYYSHWALSAGLNNAAGLGFRGYSGRG-VPRWDGVSDGDPW 303
Asian arowana          RILTGFLVSHPP---GLAP-FWAPTQVLWVWAMSVALRMRYYSHWALSDALNNAAGLGFGGHGHSG-APLWDSMSDGEPW 286
Atlantic herring       HALTIIILKVSSV---NLNN-FGGLQGVLWVWNLSLLLKMSYYSHWALSECLCNATGLGFCAKRPTG-SQRWNGLSDGELW 289
Atlantic salmon        NSLTRLLRDNAS---SLSVSNNVLTDVLWIWGLAVVLRIRYYSHWKISECLNNAAGLGFRGMVKED-SPNWSGLSDGDLW 286
Rainbow trout          NYLTRLLRDNAS---SLSVSNNVLTDVLWIWGLAVVLRIRYYSHWKISECLNNAAGLGFRGMVRED-NPNWSGLSDGDLW 286
Stickleback            CLLVHLLNACDP---S--A-SGPLCGAAWVLALGAVLRIQYYSHWRCSECLNNAAGLGFWPRCPGD-PPGWSGLSDGDFW 289
Zebra mbuna            FYLVFFLKHLIH---DF-N-PGILYGILSTWCLGLVLRIQYYSHWKISECLNNAAGFGFWEDSSGDYFSKWSGLSDGDFW 288
Tilapia                FYLVYFLKYLIH---DF-N-PGILYGILSTWCLGLVLRIQYYSHWKISECLNNAAGFGFWEDSSGDYFSKWSGLSDGDFW 288
Damselfish             WCLIYVLKHNAY---DPMD-SGVLCGVLWIWGLGLVFRIQYYSHWRISESLNNAAGFGFWEPSSGG-SSEWSGLSDGDFW 291
Yellowbelly rockcod    FYVVYLLKHSYY---DSYS-S--IYGVLWVWGLALVFRIQYYSHWRISECLNNAAGFGFWEKEPED-SPDGCKLSDGDYC 289
Yellow croaker         YCLVYFLKHNAY---NPSK-SIIIYGLLWVWCLALVLRIQYYSHWRISECLNNAAGFGFGENVPGV-SPDWSRLSDGDFW 291
Striped bass           YCLVYFLKHNIY---DPYN-SIFFYGILWVWGMAVVLRMQYYSHWRISECLNNAAGFGFWENAPGC-SPDWSRLSDGDFW 291
Elephant shark         MFLTNCIRVIEGIDQGFLY-CDVSKDILLISMIALMFRLAYYSQWLLSESLNNLVGLGFEGQAKKE-RCVCSALSDADIW 301
Frog                   SLVSVNLSFQLS----LMT-CRHLNCVCIMWTTALLFKLTYYFHWLLDESLFCAAGFLTAYH---V-DGFQVTFCDTDIW 289
Coelacanth             FLIRNKESALTI-----PQ-SYMINDIFVNWRTALLFKLTYYSHWILSESLLNTAGFGFKGYDNQG-ILVGNDLTDTDIC 293
Cock                   AWLEGWLPCMQ-----------GWASMGHMWAQALLFRLAYYSQWVLDEALLEAAGFGAAV--------EHRDLSGHDLW 267
Lizard                 NMLRGYICAQAD----QND-CSCFGCVYVLWTSALFFRLTYYSNWMLDESLFLAAGLGLELD--CA-GTAEGILMDADIW 291
Alligator              LAMRGHEGTLTS----LAY-CTRFGCVYVVWISSLLFRLAYYSHWALDESLLNAAGFGLALG--HD-P-THGDLSGTAMW 286
Wild boar              VAMRWAVSVGAG----LTD-CRQLQCVYVMWSMAGLFKLTYYSHWILDESLLQAAGFGSGFG--GS-TGEESFVPDADIW 291
Mouse                  VALRSAVSAGAG----LDD-CQRLECIYLMWSTAWLFKLTYYSHWILDDSLLHAAGFGAEAG--QG-PGEEGYVPDVDIW 291
Rat                    VALRRVVSAGAG----LDD-CQRLECIYIMWSTAGLFKLTYYSHWILDDSLLHAAGFGSEAG--QR-PGEERYVPDVDIW 291
Chimpanzee             VAVSRVVDAGAG----LTD-CQQFECIYVVWTTAGLFKLTYYSHWILDDSLLHAAGFGPELG--QS-PGEEGYVPDADIW 291
Human                  VAVSRVVDAGAG----LTD-CQQFECIYVVWTTAGLFKLTYYSHWILDDSLLHAAGFGPELG--QS-PGEEGYVPDADIW 291
Rabbit                 VAMRGLVSTGAG----LSH-CRQLECIYVMWSTAGLFKLTYYSQWILDESLLHAAGFGTGFG--QS-PGEDGYVPDADIW 291
```

Exon 3

```
Channel catfish      EIETSSNISAFARLWNRTTAAWLRRLVFHRSSKMPVLMTFCFSALWHCLYPCQV AGFLGWA-VAVLGDHKLHKHLSPRL- 365
Red pirahna          EIETSTRISAFARRWNDTTTAWLRRLVFQRCSTAPLFMTFGFSAWWHCLYPCQV LGFLGWA-TAVLGDYKLHTCIQPRL- 368
Mexican cavefish     EIESSTRISMFARRWNGTTAAWLRRLVFQRCSRAPQLMTFSFSALWHCLYPCHI VGFLGWA-IAVQGDYKLHSYIKPKL- 368
Zebrafish            VTEASSRPSVFARKWNQTTVDWLRKIVFNRTSRSPLFMTFGFSALWHCLHPCQI LGFLIWA-VTVQADYKLHRFSHPKL- 357
Common carp - 2      VTEASSRPSVFARQWNRNTAVWLQRMFFKRSNRSPLFMTFGFSAWDCLHPCQIΔ2GFIIWA-ITVQGDYKIHHFLHPKL- 369
Horned barbel - 2    VTEASSRPSVFARQWNRTTAAWLRRMVFKRSNRSSLFMTFGFSAWWHCLHPCQI LGFIIWA-ITVQGDYKIHHFLHPKL- 368
Goldfish-V1          VTEALSRPSAFARQWNRTTAVWLRRMVFKRSNRSPLFMTFLFSAWWHCLHPCQI LGFIIWS-VTVQGDYKMHHFLHPML- 335
Goldfish-V2          VTEALSRPSAFARQWNRTTAVWLRRMVFKRSNRSPLFMTFLFSAWWHCLHPCQI LGFIIWS-VTVQGDYKMHHFLHPML- 309
Horned barbel - 1    VTEALSRPSAFACQWNRTTAVWLRRMVFKRSNRSPLFMTFLFSAWWHCLHPCQI LAFSSGPSPCVQGDYKIHHFLHPML- 369
Common carp - 1      VTEALSRPSAFARQWNRTTAVWLRRMVFKRSNRSPLFMTFLFSAWWHCLHPCQI LGFIIWA-VTVQGDYKIHHFLHPML- 368
Spotted gar          TLETLTRLSHFARMWNKTTAEWLRRLVFQKCHVSPLLLTFGFSAWWHCLHPCQL VGFLCWA-ATVEADYRIHHYLSPYM- 371
Japanese eel         TLETCSRPSAFARHWNGTTAAWLRRAVFQRCRTAPLAMTFGFSAWWHCLHPCQV LGFLLWG-VAVKADYRIHQRVRPLL- 381
Asian arowana        TVERSSRLSEFARRWNGTTAAWLRRLVFQRCKVARLALTFGFSAWWHCLHPCQV LGFLVWA-AAVQADYRVHRRLRPLA- 364
Atlantic herring     TTETSCRLSEFTRQWNGTTAAWLRRLIFQRFQTFPLGLTFAFSALWHCLHPCQV AGFFLWA-TSVKADYQVHKYLSPRL- 367
Atlantic salmon      TTEMSCQVSEFARRWNGTTATWLRRLVFQRCKTAPLVMTFGFSIWWHCIHLCQF VGFLVWA-AAVRADYQIHKYLKPKL- 364
Rainbow trout        TTEMSCQVSEFARRWNGTTATWLRRLVFQRCKTAPLVMTFGFSIWWHCIHLCQF VGFLLWA-AAVRADYQIHKYLKPKL- 364
Stickleback          SVEASSRMSQFARRWNATTASWLRRLVYARCKRFPLLATFSFSLWWHCCHLCHL VGMLTWA-ATVTADRHAHGLLQPKL- 367
Zebra mbuna          TTEASICMSQFARRWNATTASWLRRLVYARHKHFPLLMCFGFSLWWHCLHLCHF VGFFTWA-ATVKADHHIHRNLLPNI- 366
Tilapia              TTEASICMSQFARRWNAMTASWLRRLVYARHKHFPLFMCFGFSLWWHCLHLCHF VGFFTWA-ATVKADHHIHRNLFPNI- 366
Damselfish           TTEASHRMSDFARRWNVTTALWLRRLVYTRCKHFPLFMSFGFSLWWHCLHLCHF VGFLTWA-ATVKADYHIHKHLGPEV- 369
Yellowbelly rockcod  TIEASSRMSEFARRWNATTASWLRRLIYIRCKRFPLFMTFSFSLWWHCCHLCHF VGMWTWA-ATVKADYHIHRYLHPKL- 367
Yellow croaker       IIEASNRMSQFARRWNATTASWLRRLVYTRCKRFPLFMTFSFSLWWHCLHLCQI VGFLTWA-ATVKADYHIHRCLWPKL- 369
Striped bass         TTEASSRISEFARRWNTTTASWLRRLIYMRCKRFPLFMTFGFSLWWHCLHLCHF VGILTWA-ATVKADYHIHGHLRRRL- 369
Elephant shark       TLETTNKISEFTRTWNKTTADWLRRMVFQRSKVHPLLATFAFSAWWHCLHPCQV FGFVLWA-CSVKADYYVHHYIMPLTK 380
Frog                 TLETTHKISVFTRTWNKSTASWLRRIIFEKCKIGSLLMTFAFSAWWHCLHPCHI FGFLCWA-LLVKADYRIHKYFNPCQ- 367
Coelacanth           TLETTNKISQFARAWNKTTAEWLRRLVFQRCTVHPLFVTFAFSAWWHCLHPCQI FGFLFWS-VAVEADKRVHYYLAPLA- 371
Cock                 TLETTHRLSVFSRTWNRSTSLWLRRLVFQRCPVQPLLATFAFSAWWHCLRPCQI FGFLCWA-IMVEADYRIHPFLSSRA- 345
Lizard               TLETTNTIAVFTRTWNKSTAQWLRRLVFQRSKSHPLWATFAFSAWWHCLHPCQV FGFLCWA-VMVKADYGFHRFFRSIA- 369
Alligator            TLETTHRIALFTRTWNRSTAQWLRRLIFQRSPTQPLLATFTFSAWWHCLYPCQV FGFLCWA-VMVEADYRIHPFLSNLT- 364
Wild boar            TLETTHKLSLFTRKWNHSTAQWLRRLVFQQGRTWPLLQTFVFSAWWHCLHPCQV FGFLCWA-VMVKADYLIHTFAKVSI- 369
Mouse                TLETTHRISLFARQWNRSTALWLRRLVFRKSRRWPLLQTFAFSAWWHCLHPCQV FGFLCWS-VMVKADYLIHTFANVCI- 369
Rat                  TLETTHRISLFARQWNRSTAQWLKRLVFQRSRRWPVLQTFAFSAWWHCLHPCQV FGFLCWS-VMVKADYLIHTFANGCI- 369
Chimpanzee           TLERTHRISVFARKWNQSTARWLRRLVFQHSRAWPLLQTFAFSAWWHCLHPCQV FGFVCWA-VMVEADYLIHSFANEFI- 369
Human                TLERTHRISVFSRKWNQSTARWLRRLVFQHSRAWPLLQTFAFSAWWHCLHPCQV FGFVCWA-VMVEADYLIHSFANEFI- 369
Rabbit               TLETTHRIALFTRKWNHSTAQWLRRLIFQRRRVWPLFQTFAFSAWWHCLHPCQV FGFLCWA-VMVEADYLIHTFANVFI- 369
```

Exon 3

```
Channel catfish      TTAWRKGLFTCLSWLYTQVVIACVVVTTELQSLEALMLFCTTHIALFPLASIL--ILFIL------------------ 423
Red pirahna          TVLWKKRLYSLLGWAYTQAVITCVVLTTELQSSDAVMLLYPTHIVVVPFVTVV--ILFIL------------------ 426
Mexican cavefish     TTPWKKGLYACVGWAYTQIVIGCIVLATELQSFVAVKLLWTTRIALVPLASVV--ILIIL------------------ 426
Zebrafish            NSLWRKRLYVCVNWAFTQLTVACVVVCVELQSLASVKLLWSSCIAVFPLLSAL--ILIIL------------------ 415
Common carp - 2      TSKWRNWLNVCLNWAFTQLTSSCV-VCVELQSLESVKLLWSXYIAVFPLLSVL--MIIIL------------------ 426
Horned barbel - 2    TSKWRKWLYVCLNWAFTQLTISCVVVCVELQSLESVELLWSSYIAVFPLLSVL--MIIIL------------------ 426
Goldfish-V1          TSKWRKWLYVCLNWAFTQLTISCVVVCVELQSLESVKLLWCSYIAVLPLLSVL--IIIIL------------------ 393
Goldfish-V2          TSKWRKWLYVCLNWAFTQLTISCVVVCVELQSLESVKLLWCSYIAVLPLLSVL--IIIIL------------------ 367
Horned barbel - 1    TSKWRKWLYVCLNWAFTQLTISCVVVCVELQSLESVKLLWSSYIAVFPLLSVL--IIIIL------------------ 427
Common carp - 1      TSKWRKWLYVCLNWAFTQLTISCVVVCVELQSLESVKLLWSSYIAVFPLLSVL--IIIIL------------------ 426
Spotted gar          DSKVKRLIYKVLTWFQTQLVIACIVVIIELRYLSSVWLLFKAYIGLFPLLNCL--VLVVLPRKQQIKVLY-------- 439
Japanese eel         TSPATQRLYRCLGWAHTQLLIACVVVAVELRGACFMWLLWKTYISVFLLLYVI--LLFFLPQNIPKNE---------- 447
Asian arowana        SSPGKRLVYTCLSWAQTQLVITWVVAVELRNISSVWLLCRSRASWFPVLHIL--LTFFMMCLV--------------- 426
Atlantic herring     TSPWTRKVFRCLSWIQTQMVMACVIVTIELRHVT-LWSFCMNYISIFPLIYIC--LLTALSFC--------------- 427
Atlantic salmon      TSTRRRLVRTCLCWLNTQMVMACVVIAIELRSTSSLRILGLTYTGVFPLLNVL--FIFIV------------------ 422
Rainbow trout        TSTRRRLVRTCLCWLNTQMVMACVVIAIELRSTSSLKILGLTYTGVFPLLNVL--FIFIV------------------ 422
Stickleback          SPAWRK-VYACLSWINTQMNVACIVTAVELRSVSGLRLLSTTYVGLVPLFNIL--LLLILLKHNTV------------ 430
Zebra mbuna          TPTQRK-IYTFVNWINTQMIVTCIVIAVEFRNSSGLRLLSKTYIGLFPLVNII--LHFIVLNLNSLEQ---------- 431
Tilapia              TPTQRK-IYTFVNWINTQMVVTCIVIAVEFRNLSGLNLLSKTYIGLFPLVNII--LLFIILNLNSLEQ---------- 431
Damselfish           SPAGRK-IYSVLGWINTQMVVTCIAIAVEFRNMSGLRLLWGTYAGLFPLANIT--LLLILIKLQRHD----------- 433
Yellowbelly rockcod  SSTQSK-IYSCLGWINTQMIVACIVIAIELRDMSGLRLLSITYIGLFPLLNIM--LLFILLKLNKV------------ 430
Yellow croaker       SSSWRK-IYTCLSWINTQMIVSCIVIAVEIRNMSSLILLSTTYIGLFPLFNII--LLFILQKLKFTKEVM-------- 436
Striped bass         SSTWRK-TYTCLSWINTQMIVACIVIAVELRNMSGLRLLSTTYIGLFPLCNII--LLFILLKLNTV------------ 432
Elephant shark       KSRLLLIVYKVFTWIQTRLITAYILIAVELRTLAYVLMMCRSACILSPVMYMLVLVAMYIVSQHH------------- 445
Frog                 QSWCTRVLYRIFTWLHTQLIVAFLIVAIEMRSIKIIWSLCLSYNCYFPILYCL--SLISSIKRK-------------- 429
Coelacanth           NSWFTKQLFKVFTWIQTQLVIACIMMAIENKSFSSLWLLSQSCIILFPLLYCL--VLLLLPKRPRI------------ 435
Cock                 TSCVAKLLYRGTTWVFTQLIIAYIQMAVETESFSMLCLLWTSYNSILPLTYGLVLLLLLFAQKPKQNRACPGLTRLAG 424
Lizard               KSQPQRVVCQSLTWCHTQLVVAYIIIAVERRTLSVLWWLFFSYNSFFPLVYVI--SLLLLAKEKAISPTDPHVHTS--- 443
Alligator            KSWCTRLLYKALTWVLTQLIIAYIMVAVEMRSFSALWLLCTSYISFFPLLYCI--SLLLLAKRATQK----------- 429
Wild boar            RSQLMWLLYRTLTWAHTQLIIAYIILAVEARSLSSLWLLCNSYNSVFPMVYCI--LFFLLAKRKHKCN---------- 435
Mouse                RSWPLRLLYRALTWAHTQLIIAYIMLAVEGRSLSSLCQLCCSYNSLFPVMYGL--LLFLLAERKDKRN---------- 435
Rat                  RSWPLRLLYRSLTWAHTQIIIAYVMLAVEGRSFSSLCRLCCSYNSIFPVTYCL--LLFLLARRKHKCN---------- 435
Chimpanzee           RSWPMRLFYRTLTWAHTQLIIAYIMLAVEVRSLSSLWLLCNSYNSVFPMVYCI--LLLLLVKRKHKCN---------- 435
Human                RSWPMRLFYRTLTWAHTQLIIAYIMLAVEVRSLSSLWLLCNSYNSVFPMVYCI--LLLLLAKRKHKCN---------- 435
Rabbit               RSWPLRLLYRTITWVHTQLIIAYIMVAVEVRSLSSLWMLGNSYSSVFPAVYCT--LLFLLAKRKHKFNFLKKIFT---- 442
```
